# Stereocomplexed Functional and Statistical Poly(lactide-carbonate)s via a Simple Organocatalytic System

**DOI:** 10.1021/acs.macromol.3c02485

**Published:** 2024-02-28

**Authors:** Panagiotis Bexis, Jonathan T. Husband, Haritz Sardon, Olivier Coulembier, Andrew P. Dove

**Affiliations:** † School of Chemistry, University of Birmingham, Edgbaston, Birmingham B15 2TT, U.K.; ‡ Center of Innovation and Research in Materials and Polymers (CIRMAP), Laboratory of Polymeric and Composite Materials, University of Mons, Mons B-7000, Belgium; § POLYMAT, University of the Basque Country UPV/EHU, Joxe Mari Korta Center, Avda. Tolosa 72, 20018 Donostia-San Sebastian, Spain

## Abstract

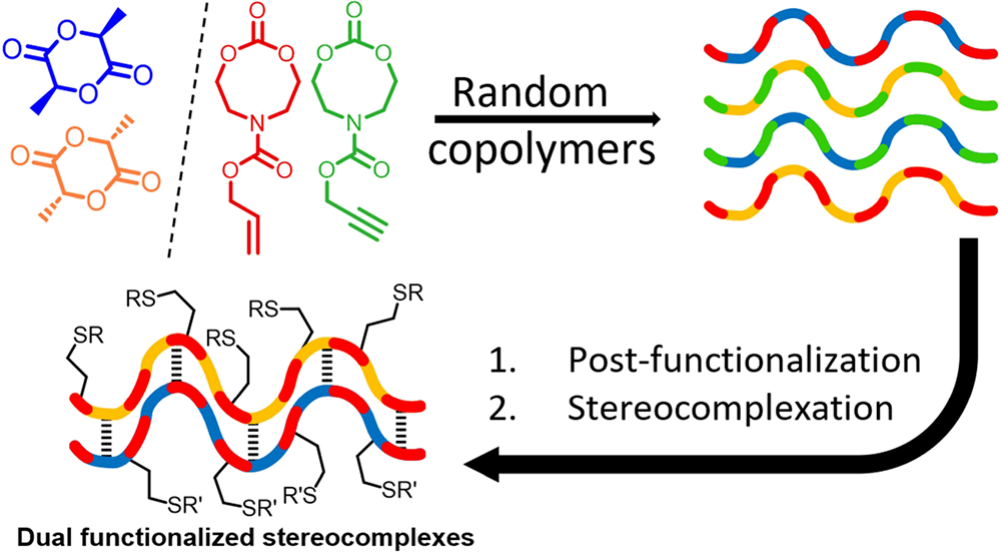

The stereocomplexation of polylactide (PLA) has been widely relied upon to develop degradable, sustainable materials with increased strength and improved material properties in comparison to stereopure PLA. However, forming functionalized copolymers of PLA while retaining high crystallinity remains elusive. Herein, the controlled ring-opening copolymerization (ROCOP) of lactide (LA) and functionalized cyclic carbonate monomers is undertaken. The produced polymers are shown to remain crystalline up to 25 mol % carbonate content and are efficiently stereocomplexed with homopolymer PLA and copolymers of opposite chirality. Polymers with alkene and alkyne pendent handles are shown to undergo efficient derivatization with thiol–ene click chemistry, which would allow both the covalent conjugation of therapeutic moieties and tuning of material properties.

## Introduction

Polyesters and polycarbonates are finding increased use in both academia and industry to realize biodegradable and biocompatible polymers for applications such as biomedical devices, tissue scaffolds, and degradable packaging.^1−4^ One of the most widespread in use is polylactic acid, or polylactide (PLA), most commonly encountered in commodity biobased packaging applications.^5^ Readily available PLA, synthesized from the polymerization of enantiomerically pure lactide, is typically a brittle material.^6^ However, it is known that the blending of enantiopure PLA homochiral chains of opposing chirality leads to stereocomplexed PLA (sc-PLA). This microstructure imparts enhanced crystallinity, and therefore, enhances the thermal and mechanical properties, in comparison to isotactic PLA.^6,7^ Stereocomplexation also enables toughening of block polymer hydrogels and elastomers as a result of the greater levels of crystallization.^8−13^

Imparting functionality into PLA allows for the selective tailoring of thermomechanical properties while also allowing the incorporation of functionality into the polymer structures.^14^ This can be readily achieved through PLA polymer blends or copolymerization of pendent-derivatized lactide monomers or other cyclic monomers with lactide. However, doing so typically leads to a marked decrease in crystallinity because of the nonrandom incorporation of the comonomers. Even at low incorporation levels, this leads to a reduced heat deflection temperature and mechanical strength.^15,16^

Recently, strained *N*-substituted 8-membered cyclic carbonates have emerged as the basis for a class of new biodegradable and biocompatible polymers.^17−22^ The *N*-substitution provides a synthetic handle to attach functionalities. Tuning the functionality of these polymers has been successfully accomplished through their post-ring opening polymerization (ROP) modification as well as through the preparation of functionalized monomers.^18,23^ With the increased ring strain of these functionalized 8-membered cyclic *N*-carbonates, in comparison to the more commonly studied 6-membered analogues, and consequent enhanced polymerization reactivity,^18,24^ it was proposed that a statistical copolymerization of these monomers with lactide could be achieved with a close to random incorporation of each monomer. In doing so, we hypothesized that a semicrystalline, functional PLA-carbonate-based material could be obtained. Moreover, we postulated that this approach would allow for stereocomplex formation alongside functional group incorporation, which would represent a significant advance in stereocomplex polymer design that could have implications in applications such as drug delivery, cellular imaging, and biological scaffolds.^25^

In this work, the copolymerization of previously described alkyne- and alkene-functionalized 8-membered cyclic carbonates (P8NC^23^ and A8NC^21^) with stereopure lactide was undertaken via ring-opening copolymerization (ROCOP). Subsequently, the thermal properties of the copolymers produced were investigated alongside their potential stereocomplexation and postpolymerization modification capabilities.

## Results and Discussion

**Figure 1:**
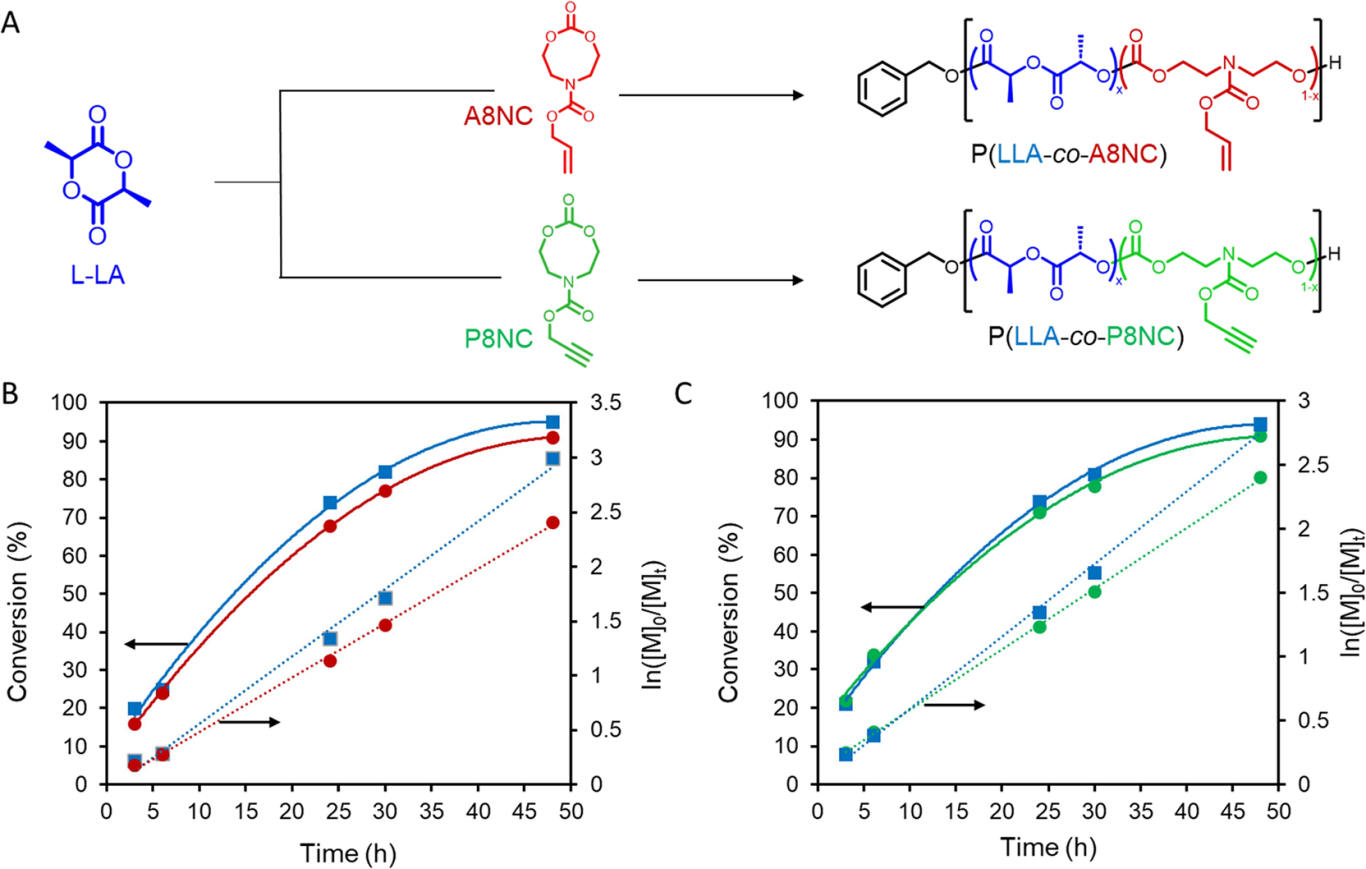
Organocatalyzed copolymerization of LLA (*blue*) with A8NC (*red*) and P8NC (*green*) comonomers; (A). Kinetics of LLA copolymerizations with (B) A8NC and (C) P8NC; ([M_tot_]_0_/[I]_0_= 50/1, [DPP]:[DMAP] = 10 mol %:20 mol % relative to [M_tot_]_0_).

Monomers propargyl 2-oxo-1,3,6-dioxazocane-6-carboxylate (P8NC) and allyl 2-oxo-1,3,6-dioxazocane-6-carboxylate (A8NC) (Figure 1) were synthesized by ring closing of the respective diols with triphosgene, as reported previously (Schemes S1, S2; Figures S1, S2).^21,23^ Following a screening of various organocatalytic systems (Figure S3; Table S1), it was found that the combination of diphenyl phosphate (DPP) and 4-dimethylaminopyridine (DMAP) proved to be the most suitable to serve the targeted copolymerization behavior on account of the even incorporation of monomers throughout the polymerization and low dispersity of the result polymers (Figure 1A).^26^ To this end, using DPP and DMAP at a ratio of 1:2, benzyl alcohol (BnOH) as initiator with a feed comonomer ratio of *f*_8NC_ = 0.25 and *f*_LLA_ = 0.75 (Table S1) revealed 95% LLA and 91% A8NC for the allyl copolymer (Figures 1B and S4) and 94% LLA and 91% P8NC for the propargyl copolymer (Figure 1C) within 48 h, as determined by ^1^H NMR spectroscopy. Studying the reactivity ratios of the alkene-functional carbonate and lactide, a small preference for LLA homopolymerization over cross-polymerization was revealed. However, the copolymerization is expected to approximate a random copolymerization (Figure S5 and supporting discussion). The copolymerization of A8NC with LLA was expanded to study the effect of carbonate feed loadings on thermal properties, with feeds (*f*_A8NC_) ranging from 10 to 90 mol % (Table S2). All polymerizations were terminated once both monomers had reached a conversion of ≥90%. The experimental molar ratio of the carbonate in the copolymers (*F*_A8NC_) was close to the feed values (*f*_A8NC_) in each case. This result, combined with the similar polymerization rate of both comonomers, suggests that carbonate monomer incorporation into the PLA chain is approximately random. In the ^1^H NMR spectra of the resultant polymers, two small peaks next to the poly-l-lactide (PLLA) methine and methyl protons are observed at δ = 5.01–5.05 ppm and δ = 1.49–1.53 ppm, respectively, and are identified as distinct lactide units (L) next to a carbonate unit (C) (Figures S6 and S7).^27^

**Figure 2:**
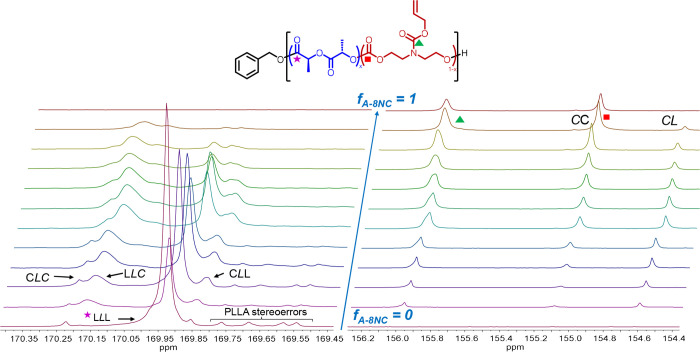
Stacked, focused ^13^C NMR spectra of the P(LLA-*co*-A8NC) copolymers of increasing carbonate content (Table S2) at the carbonyl region of the polyester (left) and polycarbonate (right).

Further analysis of the ^13^C NMR spectra revealed additional dyads as the carbonyl carbon signal is highly sensitive to its neighboring monomer (Figures 2 and S8). The PLA carbonyl carbon has a chemical shift at δ = 169.92 ppm in CDCl_3_ which is analogous to a homopolymer triad labeled (LLL). Inspecting the upfield region at δ = 169.54–169.76 ppm, resonances resulting from stereoerrors in the chiral polyester chain can be identified. Their low intensity indicates minimal epimerization of the monomer during ROP. As increasing amounts of carbonate units are incorporated, broader peaks appear upfield and downfield to the PLLA resonance.

These can be attributed as peaks arising from ester carbons adjacent with different carbonate environments (CLC, LLC, and CLL).^28^ An analogous scenario is observed in the polycarbonate carbonyl resonance. When lactide is copolymerized and incorporated, a new resonance gradually appears upfield to the carbonate carbon at δ = 154.58 ppm. This new peak is identified as a carbonate carbonyl carbon next to an ester monomer, representing a sequence dyad (CL). Also noteworthy is that when *F*_LLA_ ≥ 0.6, this sequence peak becomes larger in area than the carbonate–carbonate (CC) peak. This provides robust evidence of high ester-carbonate sequence in the copolymer, rather than a blocky copolymer with a high content of homopolymer (CC). These observations were confirmed by studying synthesized model diblock copolymers P(A8NC)-*block*-PLLA, which did not possess significant CL or LC resonances by ^1^H/^13^C NMR spectroscopy (Figures S3, S9, and S10). Diffusion-ordered NMR spectroscopy (Figure S11) proved that the statistical copolymers were diffusing as one entity and MALDI-ToF MS (Figure S12) showed the presence of both monomers in the statistical polymer chain and multiple sodium charged benzyl alcohol α-capped distributions separated by regular spacings of either 144 *m*/*z* or 215 *m*/*z* which correspond to the molar mass of the used monomers.

Satisfied to have successfully incorporated the functionalized carbonates into random copolymers with PLA, differential scanning calorimetry (DSC) was undertaken to understand the effect of the carbonate incorporation on the thermal properties of the polymers (Figure S13). DSC analysis of pure PLLA and PDLA control polymers prepared via organocatalytic ROP of l-lactide and d-lactide (Table S3, entries 13–14) showed melting peaks in solution-crystallized (precipitated) samples at 151.4 and 152.8 °C, respectively. To compare, DSC thermograms of the poly(ester-carbonate)s were also obtained and assessed. First, the series of P(LLA-*co*-A8NC) copolymers were analyzed. In this series, copolymers containing a lactide content of <76 mol % showed no *T*_m_ and polymers were found to be completely amorphous. However, for copolymers with incorporation of up to 24 mol % A8NC, a melting peak was observed, showing a semicrystalline nature (Table S3, entries 9–12, Figure S13). While such high comonomer incorporations into PLA crystals are reported in the literature for lactone-based systems, this is an unprecedented carbonate total content into a sustainable PLA crystal.^29−32^ Throughout the series, an increasing incorporation of carbonate units in the polymer led to a decrease in *T*_g_ and *T*_m_ values. In fact, plots of the melting temperature observed during the first heating scan, *T*_m1_ and *X*_c_ (%) against the experimental molar fraction of carbonate monomers against PDLA and PLLA in the samples follow a linear trend (Figure S14). This provides the opportunity to selectively predict and, therefore, tune the melting temperature of a desired copolymer based on any desired carbonate incorporation. Similar linear behavior was demonstrated for the single *T*_g_ of the materials, closely following the Fox equation (Figure S15), thus demonstrating the statisticality of the polymerization. In contrast, the P(A8NC)_30_-*block*-PLLA_30_ diblock copolymer displayed two distinct *T*_g_s attributed to the phase separation of the two immiscible blocks (Figure S16).

Extension of the same analysis to the propargyl-containing copolymers revealed comparable results to the allyl series (Tables S4 and S5). Only a single *T*_g_ could be observed for copolymers in this series, and analogous to the allyl samples, the observed glass transition temperature increased linearly with increasing LA content seamlessly overlapping the theoretical model (Figure S17). Studying the *T*_m_ of the P8NC copolymers, it was found that those with a carbonate content up to 19 mol % were able to crystallize and melt, which is slightly lower than 24 mol % carbonate incorporation observed for the A8NC series. This is attributed to a marginally higher reactivity of the P8NC, which increased the statisticality of the copolymer and hence decreased the lactidyl block lengths. It was found that P(LLA-*co*-P8NC_26%_) had an average lactidyl block length, *Ł*_LA_ of 8.8 whereas P(LLA-*co*-A8NC_24%_) had a *Ł*_LA_ of 10.9 (Figure S18, Equations S2 and S3). Both observations are consistent with literature precedent that a *Ł*_LA_ of 10 is usually required for crystals to form.^33,34^ For the whole propargylic series, measured *T*_m_ values increased linearly with an increasing PLLA content. Thermogravimetric analysis (TGA) showed that the progressive incorporation of less thermally stable carbonates into the PLA chain led to a depression of its thermal resilience (Figures S19 and S20). Interestingly, P(LLA-*co*-P8NC_14%_) exhibited a thermal decomposition onset temperature *T*_d_ of 270 °C and a *T*_max_ of 350 °C, whereas P(LLA-*co*-A8NC) of similar carbonate loading exhibited *T*_d_ and *T*_max_ about 50 °C lower.

Having shown that both copolymer blends displayed crystalline behavior at or below at least 19 mol % incorporation of functionalized carbonate, it was postulated that PLA-driven stereocomplexation of opposite chirality should be possible. This would facilitate the creation of functionalized PLA with improved thermomechanical properties and possibly allow stereocomplexation-driven CDSA (crystallization-driven self-assembly) of block copolymers, toward precise and tunable macromolecular structures.^25^ To achieve this, equimolar blends were prepared by combining solutions of the polymers with chloroform at room temperature. Precipitation into cold hexanes afforded the isolation of the desired materials. In addition to control PDLA and PLLA blends, equimolar amounts of the alkene-functional, semicrystalline poly(ester-carbonate)s series- P(LLA-*co*-A8NC_9.4%_), P(LLA-*co*-A8NC_14.1%_), P(LLA-*co*-A8NC_20.2%_) and P(LLA-*co*-A8NC_24.7%_), and opposite chirality PDLA were prepared by the same method. All blends were analyzed by DSC for a possible stereocomplexation behavior. Positively, all polymers that displayed a semicrystalline character successfully underwent stereocomplexation. Blends of P(LLA-*co*-A8NC) with PDLA exhibited stereocomplex melting temperatures from 172 to 194 °C (from first DSC heating scans). This is 60–80 °C higher than the homochiral parent polymers, characteristic of PLA stereocomplex formation.^35,36^ For all stereocomplexes, an additional annealing step (heating the polymers between the homochiral *T*_m_ and the stereocomplex *T*_m_ for 24 h *in vacuo*) was required to enhance stereocomplex formation and convert homochiral crystallites into stereocomplex.

**Figure 3:**
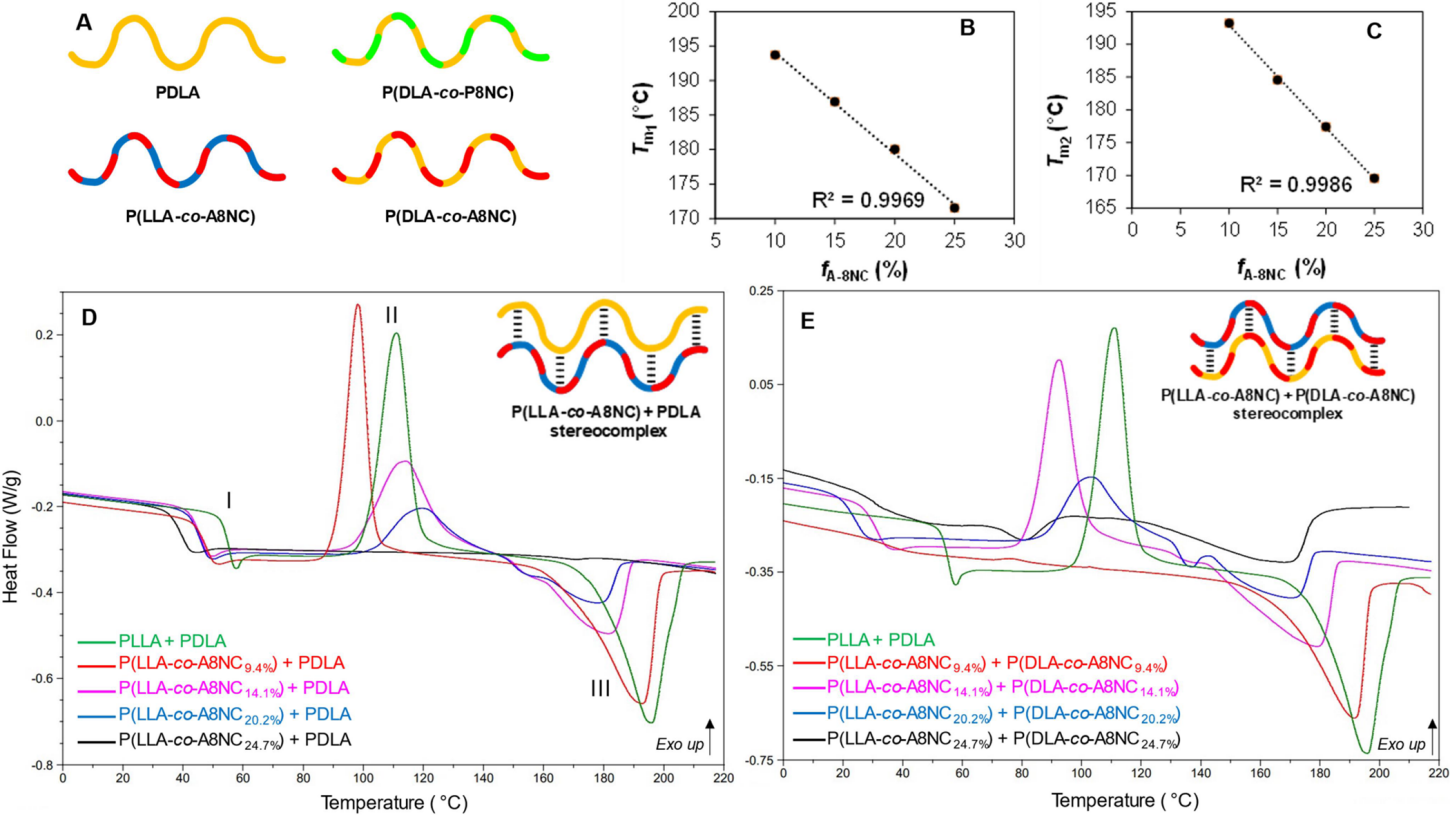
(A) Cartoon representation of available polymer and copolymer structures for stereocomplexation experiments; (B, C) Plots of *T*_m1_ and *T*_m2_ belonging to the 1st and 2nd heating scans of the equimolar stereocomplexes comprised of P(LLA-*co*-A8NC) + P(DLA-*co*-A8NC) (Table S6, entries 5–8) against the molar feed ratio (*f*) of the carbonate; Stacked DSC thermograms of equimolar stereocomplexes of (D) P(LLA-*co*-A8NC) copolymers with PDLA (Table S6, entries 1–4, postannealing 2nd heating scans), (E) equimolar stereocomplexes of P(LLA-*co*-A8NC) copolymers with P(DLA-*co*-A8NC) copolymers of identical composition, postannealing (Table S6, entries 5–8, 2nd heating scans). The thermograms display the glass transition (I), crystallization peak of the stereocomplexes (II), and the melting temperature of the resulting SCs (III).

From studying DSC thermograms of blends of P(LLA-*co*-A8NC) with (i) PDLA and (ii) their opposite chirality analogues (Figure 3A,D,E, Table S6), a decrease in the melting temperatures and a broadening of the endotherm are observed as the carbonate incorporation of the parent copolymers increased. In fact, plotting the molar feed of the carbonate against the *T*_m_ of the first and second heating runs (Figures 3B,C, S21, S22) revealed a linear relationship, with a decrease in crystallinity observed with reducing lactidyl content. As with the homopolymers, a similar reduction in the thermal stability of the materials was shown via TGA (Figures S23–S26).

It is known that although the melting enthalpy for PLLA and PDLA stereocomplexes is the greatest for equimolar blends, stereocomplexation can also occur at significantly asymmetric ratios.^37,38^ To investigate if this phenomenon could also be observed in the copolymer blends of (i) PDLA + P(LLA-*co*-A8NC_9.4%_) and (ii) blends of P(LLA-*co*-A8NC_9.4%_) + P(DLA-*co*-A8NC_9.4%_), molar mixing ratios of 95:5, 75:25, 50:50, 25:75, and 5:95 of (i), (ii) polymers were used and screened in each case. Promisingly, DSC measurements showed that a stereocomplexation melt could be observed in all blends at around 190 °C (Figures S27–S30). In the most asymmetric blends, clear homochiral melting events could also be observed at around 150 °C. For the 50:50–75:25 blending ratios of PDLA + P(LLA-*co*-A8NC_9.4%_), only the stereocomplex melting can be observed in the first heating scan. However, a second heating scan erased all homochiral melting peaks and for all ratios led to stereocomplex melting events only (Figure S28), highlighting the propensity of the blends to possess highly ordered crystalline domains. The melting enthalpy of samples varied from 1.3 to 39 J/g depending on the blending ratio, with more asymmetrical blends having lower enthalpies, as would be expected. As a control, a DSC study of the earlier synthesized diblock copolymer P(A8NC)-*block*-PLLA blended with PDLA was undertaken. After annealing at 120 °C for 24 h, the first heating scan revealed two distinct endotherms, at 141 and 186 °C with similar melting enthalpies (∼35 J/g), identified as the *T*_m_ of PDLA and the stereocomplex, respectively (Figure S31). Second heating scans showed no crystallization or melting events, indicating that optimization of recrystallization conditions is required for the homochiral melts. Extension of these studies to P8NC/LA copolymers with up to 20 mol % carbonate incorporation led to comparable results (Table S7, Figures S32–S35).^39−41^

**Figure 4:**
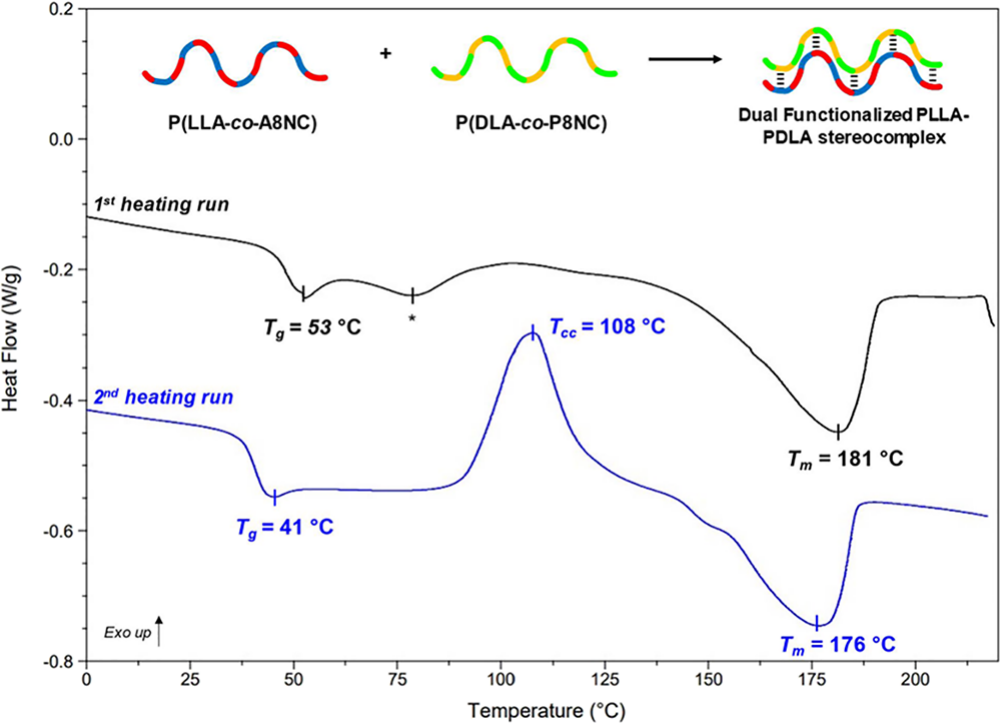
DSC thermogram of the stereocomplex consisted of an equimolar quantity of P(LLA-*co*-A8NC_9.4%_) and P(DLA-*co*-P8NC_10.7%_), with annotated *T*_g_, *T*_cc_, and *T*_m_ values. *Peak at 77 °C attributed to a homochiral melting event.

While each series was shown to form a stereocomplex to a copolymer with opposite PLA chirality, it was decided to evaluate the affinity of each functionalized copolymer to form a stereocomplex with the other. Thus, P(LLA-*co*-A8NC_9.4%_) was mixed with P(DLA-*co*-P8NC_10.7%_) at equimolar quantities, precipitated, and annealed at 120 °C. The DSC thermogram of the result blend shows a small homochiral domain in the first scan (77 °C); however, as per previous mixtures no homochiral melting was observed in the second scan (Figure 4). In the second scan, a *T*_g_ of 41 °C is followed by a cold crystallization (*T*_cc_) peak at 108 °C, with a high enthalpy of crystallization (29 J/g). This is finally followed by the stereocomplex melt at 176 °C. This material exhibits strong stereocomplexation behavior, with dual functionality (allyl and alkynyl) incorporated through up to 20 mol % carbonate in the statistical PLA copolymers. This highlights a uniquely biodegradable material with strong material properties and handles for post-ROP modification for a variety of applications.

**Figure 5:**
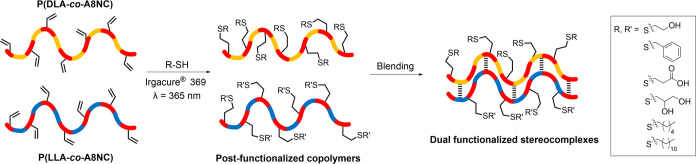
Illustration of the thiol modification of P(LA-*co*-A8NC) copolymers and subsequent stereocomplexation of opposing chirality copolymers. R and R’ reflect the possibility of different thiol functionalities within the stereocomplex crystal.

To prove crystallinity could be retained after functionalization of the pendent handles, the alkene groups were targeted for reactions with thiols by UV-mediated thiol–ene click chemistry.^42^ A range of thiols were reacted with P(LA-*co*-A8NC) copolymers of varying compositions (Figure 5, Table S8), using UV (λ = 365 nm) exposure and Irgacure 369 catalyst at 5–20 mol % respective to the pendent alkene groups (Scheme S4). An excess of thiol (5–10 equiv) was essential to achieve minimal side reactions, unimodal molar mass distribution, and full conversion as observed by Junkers and co-workers.^43^ Quantitative conversion of the terminal alkene functionality to the thiol group could be observed by ^1^H NMR spectroscopy by the disappearance of the allyl resonances at δ = 5.27–5.30 and 5.88–5.94 ppm, and the appearance of the signals of each used thiol (Figure S36). In addition, the disappearance of the alkene IR absorbance (1650 cm^–1^) and the concomitant appearance of the thiol (R-S) stretch at ca. 2325 cm^–1^ were observed, and an increase in molar mass was also observed by SEC. In addition, dispersity of the polymers was retained through the functionalization with *Đ*_M_ ≤ 1.21 (Figures S37 and S38).

**Figure 6:**
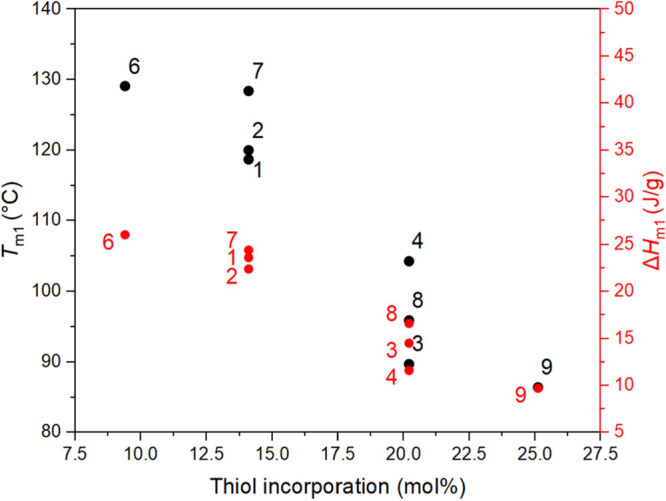
Graph showing *T*_m1_ and fusion enthalpy (Δ*H*_m_) of different P(LA-*co*-A8NC) copolymers against their thiol mol % functionalization (Table S9 for the list of used thiols and respective thermal properties).

Analysis of the thermal properties of the thiol functionalized copolymers by DSC (Table S9), at one exception, shows that samples retained their semicrystalline nature, with *T*_m_ values slightly reduced from the precursor polymers (∼10 °C lower), including when the substituent contained a protic group such as 3-mercaptopropionic acid (Figure 6). This *T*_m_ depression could also be attributed to the increase of intermolecular fractional free volume of the polymers due to the installation of larger functional groups, which could impede the ordered crystal packing and the solution-crystallization efficiency of the stereocomplex.^44^ The melt-recrystallized samples during the DSC second heating run showed an increase in *T*_m_, proving the existence of the initial imperfect crystallites.^45^ The exception, P(DLA-*co*-A8NC_25.1%_) derived with 1-thioglycerol, was completely amorphous and exhibited a *T*_g_ at 30.4 °C, hypothesized to be a result of either the structure’s chirality or because of the dual-alcohol functionality interfering with the crystal packing at this level of high functionality incorporation, >25 mol %.

Further extending this study to investigate if the copolymers could stereocomplex, equimolar blends were prepared with (i) PLAs of opposite chirality, (ii) copolymers of identical functionality and opposite chirality PLA, and, importantly, (iii) a copolymer bearing a different thiol functionality and opposite chirality. After annealing, DSC analysis showed all polymer blends had *T*_g_ values between those of the parent polymers. All blends still possessed semicrystalline properties with measured *T*_m_ values between ∼165 and 190 °C which were dependent on the carbonate incorporation and the thiol attached (Table S10). Δ*H*_m_ for the new blends exhibited the same trend, whereby the highest PLA continent blends displayed higher crystallinity and heat of fusion. Second DSC heating runs indicated that the stereocomplexes were able to recrystallize and melt again but this time at a higher temperature with greater Δ*H*_m_. This result showed that the polymers had not achieved their optimum crystallinity out of precipitation and annealing, and possibly by using optimized DSC conditions (slower cooling and heating runs), even the first run thermal results could have been improved. This result also shows that different thiol derivatives can still undergo stereocomplexation, which enables the design of dual-functionalized statistical copolymers of PLA which still undergo stereocomplexation at significant functionality incorporation (∼25 mol %).

Finally, wide-angle X-ray diffraction analysis was used to harvest more information about the stereocomplexes and conclusively validate their presence within the pre/postfunctionalized materials (Figure S39). Both pre- and postfunctionalized P(LA-*co*-A8NC) copolymers of varying carbonate content were blended with PLA of opposite chirality. The appearance of three distinct diffraction peaks belonging to PLA stereocomplexes, at 2θ = 11.95, 20.8, and 24.03° confirmed the formation of well-defined stereocomplexed domains. These are markedly different from those of 2θ = 15, 17, 19, and 22.5° for α*-* or δ(α’)*-* modifications of PLLA or PDLA homocrystallites which could not be identified in the spectrum.^46−48^

## Conclusions

Allyl and propargyl functionalized cyclic carbonates are shown to copolymerize with LA using a simple, inexpensive, and commercially available organocatalytic system to produce copolymers in which the carbonate units are randomly incorporated throughout the chain. The resultant poly(ester-carbonate) copolymers can undergo successful stereocomplexation with not only PLA but also poly(ester-carbonate) counterparts of different stereochemistry. Stereocomplexation was observed in copolymers with up to 25 mol % functionalized carbonate content, extending the range of reported levels of functional group incorporation in PLA-based copolymers. The resiliency of the system was proven through the preparation of asymmetrical molar blends of opposing chirality copolymers. Furthermore, blends containing different carbonate functionalities (propargyl and allyl) could be easily prepared. Finally, postpolymerization modification of the alkene-functionalized PLA copolymers is shown with various thiol moieties, using efficient photoinitiated thiol–ene reactions. This allows postpolymerization modification of thermal and material properties and loading of therapeutics. Thiol derived copolymers (up to ∼25 mol %) are still shown to exhibit stereocomplex formation with opposing chirality polymers, highlighting the power of this system. These findings demonstrate a method to exploit stereocomplexation behavior in PLA-based copolymers while introducing functional groups into the polymer material and will allow new opportunities for creating robust, functional biodegradable materials. The potential orthogonal installation of bioactive moieties combined with a tunable stereocomplex composition could allow a range of hydrolytic degradation profiles and targeted uses (e.g., hydrogels or carriers of bioactive load with controlled release profiles). Another potential application is the use of these materials as components of novel thermoplastic elastomers (their soft/hard character can be controlled based on the composition and the stereochemistry), and the reactive handles can also be exploited to tune these properties as well. Finally, the ability to control crystallinity through stereochemical purity or stereocomplexation can also lead to opportunities to control self-assembly processes. Examples include crystallization-driven self-assembly, which can affect particle morphology and dimensions or control the formation of higher-order structures with different properties.

## Supporting Information Available

Full synthetic and technical procedures for the monomers and polymers, including material characterization (DOCX)
