# Supplementary material for: Stereocomplexed Functional and Statistical Poly(lactide-carbonate)s via a Simple Organocatalytic System
Source: Macromolecules. 2024 Feb 28;57(5):2287–94. doi: 10.1021/acs.macromol.3c02485 (PMC10938874; doi:10.1021/acs.macromol.3c02485)
Supplement: Supplementary file 1 — ma3c02485_si_001.docx [file ma3c02485_si_001.docx]

**Supplementary Information**

Stereocomplexed Functional & Statistical Poly(lactide-carbonate)s *via* a Simple Organocatalytic System

Panagiotis Bexis,^a^ Jonathan T. Husband,^a^ Haritz Sardon,^c^ Olivier Coulembier^b*^ and Andrew P. Dove^a^*^*^*.

^a^School of Chemistry, University of Birmingham, Edgbaston, Birmingham B15 2TT, UK;

^b^Center of Innovation and Research in Materials and Polymers (CIRMAP), Laboratory of Polymeric and Composite Materials, University of Mons, Mons B-7000, Belgium

^c^ POLYMAT, University of the Basque Country UPV/EHU, Joxe Mari Korta Center, Avda. Tolosa 72, 20018 Donostia-San Sebastian, Spain

# Contents

[Materials 2](#_Toc149413418)

[Instrumentation 2](#_Toc149413419)

[General Considerations 2](#_Toc149413420)

[NMR spectroscopy 2](#_Toc149413421)

[Mass spectrometry 2](#_Toc149413422)

[Size exclusion chromatography 3](#_Toc149413423)

[FT-IR spectroscopy 3](#_Toc149413424)

[UV light source for post-polymerisation modifications 3](#_Toc149413425)

[Thermal analysis 3](#_Toc149413426)

[Synthetic procedures 4](#_Toc149413427)

[Synthesis of allyl bis(2-hydroxyethyl)carbamate (1). 4](#_Toc149413428)

[Synthesis of allyl 2-oxo-1,3,6-dioxazocane-6-carboxylate (A8NC). 4](#_Toc149413429)

[Synthesis of prop-2-yn-1-yl bis(2-hydroxyethyl)carbamate (2).^4^ 7](#_Toc149413430)

[Synthesis of prop-2-yn-1-yl 2-oxo-1,3,6-dioxazocane-6-carboxylate (P8NC).^4^ 7](#_Toc149413431)

[General procedure for the ROCOP of P8NC with L-lactide, [P8NC + L-LA]_0_ = 3.0 M, [P8NC + L-LA]_0_/[BnOH]_0_/[DPP]_0_/[DMAP]_0_ = (25+25)/1/5/10, 25 °C. 9](#_Toc149413432)

[General procedure for the ROCOP of A8NC with L-lactide, [A8NC + L-LA]_0_ = 3.0 M, [A8NC + L-LA]_0_/[BnOH]_0_/[DPP]_0_/[DMAP]_0_ = (25+25)/1/5/10, 25 °C. 9](#_Toc149413433)

[General procedure for all the stereocomplexation experiments 10](#_Toc149413434)

[General procedure for the post-ROP photo-initiated thiol-ene modifications of P(LLA-*co*-A8NC) or P(DLA-*co*-A8NC) copolymers with monofunctional thiols 10](#_Toc149413435)

[Analysis and supporting discussion 11](#_Toc149413436)

[References 40](#_Toc149413437)

# Materials

All chemicals and solvents, unless otherwise stated, were ordered from Sigma-Aldrich, Fisher Scientific and Alfa Aesar, and used without further purification. Silica gel (pore size = 40 Å) was obtained from Fischer Scientific and used as received. Dry solvents were obtained by purification over an Innovative Technology SPS alumina column and degassed by repeated freeze-pump-thawing prior to use. 3-Mercaptopropionic acid was ordered from Alfa Aesar and used as received. 1,8-Diazabicyclo[5.4.0]undec-7-ene (DBU) and benzyl alcohol (BnOH) were dried over CaH_2_, distilled, and stored under an inert atmosphere of N_2_. CDCl_3_ and C_6_D_6_ were dried over 3 Å molecular sieves, distilled and stored under an inert atmosphere of N_2_. 1-(3,5-Bis(trifluoromethyl)phenyl)-3-cyclohexylthiourea (TU)^1^ was synthesized as previously reported and dried over CaH_2_ in dry tetrahydrofuran. TU was isolated by removal of the drying agent *via* cannula filtration, followed by removal of the solvent under reduced pressure, and stored under an inert atmosphere of N_2_. 4-(Dimethylamino)pyridine (DMAP) and diphenyl phosphate (DPP) were both recrystallized from dry CH_2_Cl_2_/n-hexane and dried thoroughly under high vacuum before storage in a N_2_-filled glovebox. DMAP was also sublimed twice under high vacuum before insertion into the glovebox. L- and D- lactide (Corbion/Purac) were recrystallized from dry toluene (60 °C) three times, followed by sublimation (two times) under high vacuum (60 °C, 0.01 mbar) before insertion and storage into a N_2_-filled glovebox. Acidic Amberlyst A15 was washed repeatedly with methanol and air-dried prior to use. Irgacure 369 photo initiator was obtained from BASF and stored in a light-free environment prior to use. The PLLA and PDLA used for stereocomplexation studies were prepared by the ROP of the respective enantiomer of lactide catalyzed by DBU^2^ (CHCl_3_ SEC, RI, PS standards: *M*_n_ = 20.0 kDa, *Đ*_M_ = 1.06).

# Instrumentation

## General Considerations

All polymerizations were performed under an inert nitrogen atmosphere in a glovebox unless otherwise stated. All other chemical manipulations were performed using standard Schlenk-line techniques.

###

### NMR spectroscopy

NMR spectra were recorded on a Bruker Avance III 400 MHz, Avance III HD 400 MHz or Avance III HD 500 MHz spectrometer at 293 K unless stated otherwise. Chemical shifts are reported as *δ* in parts per million (ppm) and referenced to the residual solvent signal (CDCl_3_: ^1^H, *δ* = 7.26 ppm, ^13^C, *δ* = 77.36 ppm, (CD_3_)_2_SO: ^1^H, *δ* = 2.50 ppm, ^13^C, *δ* = 39.52 ppm, (CD_3_)_2_CO: ^1^H, *δ* = 2.05 ppm, ^13^C, *δ* = 29.84 ppm (CD_3_), 206.26 ppm (CO).

### Mass spectrometry

High resolution mass spectrometry was performed on a Bruker UHR-Q-ToF MaXis spectrometer with electrospray ionization. MALDI-ToF (matrix-assisted laser desorption ionization-time of flight) mass spectrometry analysis was performed on a Bruker Daltonics Ultraflex II mass spectrometer using a nitrogen laser delivering 2 ns pulses at 337 nm with positive ion ToF detection performed using an accelerating voltage of 25 kV. Trans-2-[3-(4-tertbutylphenyl)-2-methyl-2-propylidene]malonitrile (DCTB) was used as a matrix (0.6 μL of a 10 g L-1 solution in tetrahydrofuran), with sodium trifluoroacetate used as a cationization agent (0.6 μL of a 10 g L^-1^ solution in tetrahydrofuran). Analyte (0.3 μL of a 5 g L^-1^ solution in tetrahydrofuran) was applied in between separate loadings of DCTB and sodium trifluoroacetate, with solvent being allowed to evaporate between applications, to form a thin matrix-analyte-matrix film. All samples were measured in reflectron mode and calibrated against a 2000-5000 g mol^-1^ poly(ethylene glycol) standard. MALDI-ToF mass spectra of higher molar mass samples were recorded using a Waters QToF Premier mass spectrometer equipped with a Nd:YAG (third harmonic) operating at 355 nm with a maximum output of 65 µJ delivered to the sample in 2.2 ns pulses at 50 Hz repeating rate. Time-of-flight mass analyses were performed in the reflectron mode at a resolution of about 10,000. All the samples were analyzed using trans-2-[3-(4-tert-butylphenyl)-2-methylprop-2-enylidene]malononitrile (DCTB) as matrix. That matrix was prepared as 40 mg.mL^-1^ solution in CHCl_3_. The matrix solution (1 μL) was applied to a stainless-steel target and air-dried. Polymer samples were dissolved in THF to obtain 1 mg.mL^-1^ solutions and 50 µL of 2 mg.mL^-1^ NaI solution in acetonitrile has been added to the polymer solution. Therefore, 1 μL of this solution was applied onto the target area already bearing the matrix crystals, and air-dried. For the recording of the single-stage MS spectra, the quadrupole (rf-only mode) was set to pass all the ions of the distribution, and they were transmitted into the pusher region of the time-of-flight analyzer where they were mass analyzed with 1s integration time.

### Size exclusion chromatography

Size exclusion chromatography (SEC) was conducted on systems composed of a Varian 390-LC-Multi detector suite fitted with differential refractive index (RI), light scattering, and ultraviolet detectors, equipped with a guard column (Varian Polymer Laboratories PLGel 5 μM, 50 × 7.5 mm) and two mixed D columns (Varian Polymer Laboratories PLGel 5 μM, 300 × 7.5 mm). The mobile phase was either CHCl_3_ (HPLC grade) with 0.5% triethylamine, or tetrahydrofurane (THF) (HPLC grade with 0.5% w/w Et_3_N), with a flow rate of 1.0 mL min^-1^. SEC samples were calibrated against either Varian Polymer Laboratories Easi-Vials linear poly(styrene) standards (162 – 2.4 × 10^5^ g mol^-1^) (CHCl_3_ SEC), or linear poly(methylmethacrylate) standards (556 – 1.8 × 10^6^ g mol^-1^) (THF SEC) using Cirrus v3.3 software.

###

### FT-IR spectroscopy

IR spectra were obtained using a Perkin-Elmer Spectrum 100 FT-IR spectrometer. Observed spectra were an accumulation of 16 scans with a background scan subtracted.

###

### UV light source for post-polymerisation modifications

Photoinitiated post-polymerization functionalization were carried out in a Metalight QX1 light box equipped with 12 × 9 W bulbs with a peak output at *λ* = 365 nm. Samples were typically placed 10 cm away from the source with the bulbs arranged concentrically around them.

### Thermal analysis

Differential scanning calorimetry (DSC) and thermogravimetric analysis (TGA) were performed using Mettler Toledo DSC1 Star and TGA/DSC Star systems. DSC heating and cooling cycles were run in duplicate in series between -50 and 220 °C under a nitrogen atmosphere at a heating rate of +/- 10 °C min^-1^ in a 40 μL aluminum crucible. TGA was conducted between 25 and 500 °C at a heating rate of 10 °C min^-1^ in a 40 μL aluminum crucible.

### Wide-Angle X-ray Diffraction

Wide Angle X-ray Scattering (WAXS) was performed on a Panalytical Empyrean utilising Cu radiation (Kα1 and Kα2 radiation (l = 1.5406 Å and 1.5444 Å respectively)) and equipped with a Pixel Medipix 3D detector. Powdered samples were prepared onto low background Si-wafer sample holders and standard “powder” 2θ–θ diffraction scans were carried out in the angular range from 4° to 70° 2θ at room temperature.

# Synthetic procedures

**Scheme S1.** Two-step synthesis of allyl 2-oxo-1,3,6-dioxazocane-6-carboxylate (A8NC) monomer.

## Synthesis of allyl bis(2-hydroxyethyl)carbamate (1).

A 1 L round-bottom flask was charged with a magnetic stir bar, diethanolamine (1.1 equiv., 0.11 mol, 11.6 g), potassium carbonate (2.1 equiv., 0.21 mol, 29 g), deionized H_2_O (280 mL), and THF (140 mL). The reaction mixture was stirred for 30 min in an ice bath. Afterwards, allyl chloroformate (1 equiv., 0.1 mol, 10.6 mL) was added in one-shot directly into the reaction mixture. The reaction was kept in the ice bath for two hours stirring, and then taken out to stir at room temperature overnight. The product was extracted from ethyl acetate (3 × 300 mL) and the organic fraction was dried over Na_2_SO_4_ and later concentrated under vacuum. The product was a colorless viscous liquid (16.2 g, 89% yield). Characterization data was in accordance with what previously reported.^3^

**^1^H NMR (400 MHz, CDCl_3_):** δ 5.97 – 5.83 (CH_2_C*H*=CH_2_, 1H, ddt, *J* = 16.5, 10.7, 9.7 Hz), 5.30 – 5.17 (CH=C*H_2_,* 2H, (dd, *J* = 16.5, 1.3 Hz), (dd, *J* = 10.7, 1.3 Hz)), 4.58 – 4.56 (OC*H_2_*CH=CH_2_, d, *J* = 9.7 Hz, 2H), 4.13 (CH_2_O*H*, s, 2H), 3.77 (NCH_2_C*H_2_*OH, t, *J* = 6.1 Hz, 4H), 3.46 (NC*H_2_*CH_2_, t, J = 6.1 Hz, 4H).

**^13^C NMR (125 MHz, CDCl_3_):** *δ* 157.01 (N*C*=O), 132.97 (CH_2_*C*H=CH_2_), 117.94 (CH=*C*H_2_), 66.54 (*C*H_2_CH=CH_2_), 61.96 (CH_2_*C*H_2_OH), 52.87 (*C*H_2_CH_2_OH).

**FTIR-ATR:** 3376 cm^-1^ (O–H alcohol, stretch, broad), 1671 cm^-1^ (C=O carbamate, stretch, sharp), 1648 cm^-1^ (C=C alkene, stretch, short sharp).

**CHN Analysis:** Calculated for C_8_H_15_NO_4_: C 50.78%, H 7.99%, N 7.40% − Found: C 50.65%, H 7.89%, N 7.35%.

**MS (ESI, +ve):** (*m/z*) Calculated for [M+Na]^+^ adduct: 212.09 – Found: 212.19.

## Synthesis of allyl 2-oxo-1,3,6-dioxazocane-6-carboxylate (A8NC).

A 1L round-bottom flask was charged with a stirrer bar, allyl bis(2-hydroxyethyl)carbamate (1 equiv., 41.96 mmol, 7.94 g), triethylamine (2.6 equiv., 108 mmol, 15 mL), and 300 mL of THF. An additional funnel was affixed to the round bottom flask and charged with a solution of triphosgene (0.42 equiv., 17.5 mmol, 5.19 g) in 50 mL of THF. The setup was then placed in a liquid nitrogen and acetone bath, and the triphosgene solution was added dropwise for over 30 min. A white precipitate *(triethylamine hydrochloride)* was immediately formed upon the addition of the triphosgene. After the addition of triphosgene was completed, the reaction mixture was taken out of the liquid nitrogen-acetone bath and allowed to stir at room temperature for an additional two hours. The precipitated triethylamine hydrochloride salt was filtered away and the remaining solution was concentrated under vacuum to afford a dark viscous amber oil. This oil was then purified via flash chromatography over silica gel in an eluent consisting of methylene chloride: ethyl acetate (70 : 30 %v/v, *R*f = 0.6) to give the pure product as a colorless oil which crystallized upon standing on the bench overnight. Repeated recrystallization from hot hexane or a mixture of cold THF/warm Et_2_O afforded white needle-like crystals (3.76 g, 42% yield). Characterization data was in accordance with what previously reported.^3^

**^1^H NMR (400 MHz, CDCl_3_):** *δ(ppm) =* 5.95 – 5.87 (CH_2_C*H*=CH_2_, 1H, ddt, *J* = 16.5, 10.7, 9.7 Hz), 5.31 – 5.21 (CH=C*H_2_,* (dd, *J* = 16.5, 1.3 Hz), (dd, *J* = 10.7, 1.3 Hz)), 4.63 – 4.61 (OC*H_2_*CH=CH_2_, d, *J* = 9.7 Hz, 2H), 4.40 – 4.36 (C=OOC*H_2_*, ddd, J = 14.5, 3.7, 2.0 Hz, 4H), 3.65 – 3.60 (NC*H_2_*CH_2_OO=C, ddd, J = 14.9, 10.0, 2.0 Hz, 4H).

**^13^C NMR (125 MHz, CDCl_3_):** *δ(ppm) =* 155.54 (N*C*=O), 155.01 (O*C*=OO), 133.04 (CH_2_*C*H=CH_2_), 116.98 (CH=*C*H_2_), 68.50 (C=OO*C*H_2_, d, J = 98.9 Hz), 65.68 (O*C*H_2_CH=CH_2_), 47.91 (N*C*H_2_CH_2_OC=O d, J = 20.6 Hz).

**FTIR-ATR:** 3076 cm^-1^ (=C–H vinyl, stretch, sharp), 1752 cm^-1^ (C=O carbonate, stretch, sharp), 1695 cm^-1^ (C=O carbamate, stretch, sharp), 1648 cm^-1^ (C=C alkene, stretch, sharp).

**Figure S1.** ^1^H NMR spectra (CDCl_3_, 400 MHz) of A) purified allyl-functional diethanolamine precursor; B) allyl 2-oxo-1,3,6-dioxazocane-6-carboxylate (A8NC); C) ^13^C NMR spectrum of A8NC monomer (CDCl_3_, 125 MHz). (*CDCl_3_).

**Scheme S2.** Synthesis of prop-2-yn-1-yl 2-oxo-1,3,6-dioxazocane-6-carboxylate (P8NC).

## Synthesis of prop-2-yn-1-yl bis(2-hydroxyethyl)carbamate (2).^4^

A 1 L round-bottom flask was charged with a magnetic stir bar, diethanolamine (1.1 equiv., 0.11 mol, 11.6 g), potassium carbonate (2.1 equiv., 0.21 mol, 29 g), deionized H_2_O (280 mL), and THF (140 mL). The reaction mixture was stirred for 30 min in an ice bath. Afterwards, propargyl chloroformate (1 equiv., 0.1 mol, 9.8 mL) was added in one-shot directly into the reaction mixture. The reaction was kept in the ice bath for two hours stirring, and then taken out to stir at room temperature overnight. The product was extracted from ethyl acetate (3 × 300 mL) and the organic fraction was dried over NaSO_4_ and later concentrated under vacuum. The product was a colorless viscous oil (15.9 g, 87% yield).

**^1^H NMR (400 MHz, CDCl_3_):** *δ(ppm) =* 4.69 (C*H_2_*C≡CH, d, *J* = 2.5 Hz, 2H), 4.11 (CH_2_CH_2_O*H*, s, 2H), 3.81 – 3.76 (CH_2_C*H_2_*OH t, 4H, *J* = 6.17 Hz), 3.47 (C*H_2_*CH_2_OH, t, *J* = 6.2 Hz, 4H), 2.47 (CH_2_C≡C*H* , t, *J* = 4.69 Hz, 1H).

**^13^C NMR (126 MHz, CDCl_3_):** *δ(ppm) =* 156.29 (N*C*=O), 78.51 (CH_2_C≡*C*H), 77.61, 77.56, 77.36, 77.11, 75.08, 62.54, 61.94, 61.57, 60.51, 53.39, 52.97, 52.30, 47.10, 45.99, 30.63, 0.29.

**FTIR-ATR:** 3279 cm^-1^ (O–H alcohol, stretch, broad), 2929 cm^-1^ (O–H alcohol, stretch, broad), 2121 cm^-1^ (C≡C alkyne, stretch, weak, sharp), 1671 cm^-1^ (C=O carbamate, stretch, sharp).

**CHN Analysis:** Calculated C 60.86%, H 5.84%, N 10.14% − Found: C 60.81%, H 5.80%, N 9.94%.

**MS (ESI, +ve):** (*m/z*) Calculated for [M+Na]^+^ adduct: 212.09 – Found: 212.19.

## Synthesis of prop-2-yn-1-yl 2-oxo-1,3,6-dioxazocane-6-carboxylate (P8NC).^4^

A 1 L round-bottom flask was charged with a stir bar, prop-2-yn-1-yl bis(2-hydroxyethyl)carbamate (1 equiv., 43 mmol, 8.05 g), triethylamine (2.6 equiv., 108 mmol, 15 mL), and 300 mL of THF. An additional funnel was affixed to the round bottom flask and charged with a solution of triphosgene (0.42 equiv., 17.5 mmol, 5.19 g) in 50 mL of THF. The setup was then placed in a liquid nitrogen and acetone bath, and the triphosgene solution was added dropwise for over 30 min. A white precipitate (triethylamine hydrochloride) was immediately formed upon the addition of the triphosgene. After the addition of triphosgene was completed, the reaction mixture was taken out of the liquid nitrogen-acetone bath and allowed to stir at room temperature for an additional 2 h. The precipitated triethylamine hydrochloride salt was filtered away and the remaining solution was concentrated under vacuum to afford a tacky white solid. Repeated recrystallization from hot hexane (or heptane) or from a mixture of warm THF/cold Et_2_O afforded white needle-like crystals (4.00 g, 43.6% yield).

**^1^H NMR (400 MHz, CDCl_3_):** *δ(ppm) =* 4.73 – 4.72 (OC*H_2_*C≡CH, d, *J* = 2.5 Hz, 2H), 4.41 – 4.37 (C*H_2_*OCOO, ddd, *J* = 14.5, 3.7, 2.0 Hz, 4H), 3.66 – 3.60 (NC*H_2_*CH*_2_*, ddd, *J* = 17.5, 4.9 Hz, 4H), 2.48 (t, *J* = 2.4 Hz, 1H).

**^13^C NMR (125 MHz, CDCl_3_):** *δ(ppm) =* 155.87 (N*C*=O), 155.04 (O*C*=OO), 78.09 (C≡*C*H), 75.42 (*C*≡CH), 69.69 (*C*H*_2_*CH*_2_*NC=O), 69.32 (*C*H*_2_*CH*_2_*NC=O), 53.99 (O*C*H*_2_*C≡CH), 50.33 (*C*H*_2_*NC=O), 49.65 (*C*H*_2_*NC=O).

**FTIR-ATR:** 3076 cm^-1^ (≡C–H terminal alkyne, stretch, sharp), 2121 cm^-1^ (C=C alkyne, stretch, sharp), 1738 cm^-1^ (C=O carbonate, stretch, sharp), 1697 cm^-1^ (C=O carbamate, stretch, sharp).

**CHN Analysis:** Calculated for C_9_H_9_NO_5_: C 50.71%, H 5.20%, N 6.57% − Found: C 50.45%, H 5.29%, N 6.48%.

**MS (ESI, +ve):** (*m/z*) Calculated for [M+Na]^+^ adduct: 238.07 – Found: 238.08.

**Figure S2.** ^1^H NMR spectra (CDCl_3_, 400 MHz) of A) purified propargyl-functional diethanolamine precursor; B) prop-2-yn-1-yl 2-oxo-1,3,6-dioxazocane-6-carboxylate (P8NC); C) ^13^C NMR spectrum of P8NC monomer (CDCl_3_, 125 MHz). (*CDCl_3_).^4^

## General procedure for the ROCOP of P8NC with L-lactide, [P8NC + L-LA]0 = 3.0 M, [P8NC + L-LA]0/[BnOH]0/[DPP]0/[DMAP]0 = (25+25)/1/5/10, 25 °C.

Inside a glovebox 0.320 g (1.5 mmol) P8NC and 0.218 g (1.5 mmol) L-LA were charged into an oven-dried vial containing a stir bar. In a second vial 0.075 g (0.3 mmol) DPP and 0.073 g (0.6 mmol) DMAP were charged and dissolved in 0.9 mL dry CDCl_3_. This solution was transferred with a Pasteur pipette into the monomer containing vial and the vial was stirred vigorously until the dissolution of the monomers. From a prepared stock solution, benzyl alcohol (6.5 mg, 0.06 mmol) in 0.1 mL dry CDCl_3_ was added, and the polymerization solution was stirred vigorously for 30 sec. At predetermined time intervals, aliquots of the polymerization solution (20 µL) were withdrawn for ^1^H NMR spectroscopy (for conversion measurement) and SEC analysis (for the determination of *M*_n_ and *Đ*_M_) after being quenched with one drop of trifluoroacetic acid. At 48 h of reaction time, the polymerization was quenched with the addition of 1-2 drops of trifluoroacetic acid and the polymer was precipitated 3 times from cold hexanes and once from cold MeOH. *(Alternatively: to remove the unreacted monomers and catalysts the polymerization was quenched with 1.0 mL of acidified acetone and passed through a small plug of silica using 100% acetone as eluent, followed by removal of the solvent under vacuum. Then the polymer is dissolved in the minimum amount of methylene chloride and precipitated once from ice-cold n-hexane).* The polymer was dried in a vacuum oven at 60 °C overnight. ^1^H/^13^C NMR spectroscopy and SEC analysis were performed to determine the final characteristics of the polymer (Yield: 88%).

**^1^H NMR (400 MHz, CDCl_3_):** *δ(ppm) =* 7.46 – 7.28 (aromatics, m), 5.30 (benzyl, s), 5.23 – 5.11 (PLA methine, dq, *J* = 10.9, 7.1 Hz), 5.07 – 4.97 (PLA methine, q, *J* = 7.0 Hz), 4.79 – 4.63 (PLA methine, d, *J* = 2.4 Hz), 4.35 – 4.19 (OC*H_2_*CH_2_O, d, *J* = 8.3 Hz), 3.72 – 3.46 (OCH_2_C*H_2_*O, q, *J* = 7.3 Hz), 2.58 – 2.34 (C≡C*H*, s, 1H), 1.67 – 1.52 (PLA methyl, m, 34H), 1.53 – 1.38 (PLA methyl, m, 7H).

**^13^C NMR (125 MHz, CDCl_3_):** *δ(ppm) =* 169.94 (PLA carbonyl), 155.37 (P8NC carbamate), 154.59 (P8NC carbonate), 128.96-128.58 (aromatics), 78.34 (*C*≡CH), 75.25 (C≡*C*H), 71.85 (benzyl), 69.34 (PLA methine), 66.80-63.70 (*C*H_2_CH_2_N), 53.63 (*C*H_2_C≡CH), 47.81-46.93 (CH_2_*C*H_2_N), 16.99 (PLA methyl).

**SEC (CHCl_3_, RI):** *M*_n_ **=** 9,000 g mol^-1^ , *M*_w_ **=** 10,000 g mol^-1^, *Đ*_M_ **=** 1.11.

## **General procedure for the ROCOP of A8NC with L-lactide, [A8NC + L-LA]_0_ = 3.0 M, [A8NC + L-LA]_0_/[BnOH]_0_/[DPP]_0_/[DMAP]_0_ = (25+25)/1/5/10, 25 °C**.

Inside a glovebox 0.322 g (1.5 mmol) A8NC and 0.216 g (1.5 mmol) L-LA were charged into an oven-dried vial containing a stir bar. In a second vial 0.075 g (0.3 mmol) DPP and 0.073 g (0.6 mmol) DMAP were charged and dissolved in 0.9 mL dry CDCl_3_. This solution was transferred with a pipette into the monomer containing vial and it was stirred vigorously until the dissolution of the monomers. From a prepared stock solution, benzyl alcohol (6.5 mg, 0.06 mmol) in 0.1 mL dry CDCl3 was added, and the polymerization solution was stirred vigorously for 30 s. At predetermined time intervals, aliquots of the polymerization solution (20 µL) were withdrawn for **^1^**H NMR spectroscopy (for conversion measurement) and SEC analysis after being quenched with one drop of trifluoroacetic acid. At the desired time interval, the polymerization was quenched with the addition of 1-2 drops of trifluoroacetic acid and the polymer was precipitated 3 times from cold hexanes and once from cold MeOH. *(Alternatively: to remove the unreacted monomers and catalysts the polymerization was quenched with 1.0 mL of acidified acetone and passed through a small plug of silica using 100% acetone as eluent, followed by removal of the solvent under vacuum. Then the polymer is dissolved in the minimum amount of methylene chloride and precipitated once from ice-cold n-hexane).* The polymer was dried in a vacuum oven at 60 °C overnight. ^1^H/^13^C NMR spectroscopy and SEC analysis were performed to determine the final characteristics of the polymer (Yield: 89%).

**^1^H NMR (400 MHz, CDCl_3_):** *δ(ppm) =* 7.38 – 7.28 (aromatics, m), 5.96 – 5.84 (OCH2C*H*=CH2, ddt, J = 16.3, 10.7, 5.5 Hz), 5.31 – 5.24 (OCH2CH=C*H2*, d, J = 17.3 Hz), 5.23 – 5.11 (PLA methine, m), 5.05 – 4.97 (PLA methine, q, J = 7.1 Hz), 4.63 – 4.52 (OC*H2*CH=CH2, d, J = 5.6 Hz), 4.37 – 4.17 (OC*H2*CH2N, dd, J = 17.4, 7.6 Hz), 3.66 – 3.51 (OCH2C*H2*N, dq, J = 13.8, 7.4, 6.2 Hz), 1.60 – 1.54 (PLA methyl, dd, J = 7.3, 3.6 Hz), 1.53 – 1.46 (PLA methyl, m).

**^13^C NMR (126 MHz, CDCl_3_):** *δ (ppm) =* 169.89 (PLA carbonyl), 155.94 (N*C*=O), 155.07-154.58 (P8NC carbonyl), 132.86 (OCH_2_*C*H=CH_2_), 128.92-128.54 (aromatics), 118.12 (OCH_2_CH=*C*H_2_), 71.74 (benzyl), 69.30 (C=O*C*HO), 66.73 (O*C*H_2_CH=CH_2_), 63.76-63.45 (O*C*H_2_CH_2_O), 47.64-46.92 (OCH_2_*C*H_2_O), 16.95 (OCH*C*H_3_).

**SEC (CHCl_3_, RI):** *M*_n_ **=** 11,300 g mol^-1^, *M*_w_ **=** 11,900 g mol^-1^, *Đ*M **=** 1.05.

## General procedure for all the stereocomplexation experiments

Symmetrical or asymmetrical molar quantities (20-100 mg) of enantiomerically opposite polymers were charged into separate vials. The polymers were dissolved in CHCl3 (*ca.* 100 mg mL^-1^), blended into a single vial, and stirred for 10 min. Subsequently, the stereocomplexed polymers were precipitated from ice-cold *n*-hexane *via* dropwise addition. After careful decantation of the supernatant, the stereocomplex was dried in a vacuum oven at 60 °C overnight.

***Annealing of the stereocomplexes was done in a vacuum oven at the desired temperature. The polymers were either annealed in the DSC pan or in glass vials. The DSC or TGA analysis was done directly after the annealing.***

## General procedure for the post-ROP photo-initiated thiol-ene modifications of P(LLA-*co*-A8NC) or P(DLA-*co*-A8NC) copolymers with monofunctional thiols

P(LA-*co*-A8NC) (100 mg, *M*_n, NMR_ = 8700 g mol^-1^, 11.5 µmol) and 1-dodecanethiol (10 equivalents per alkene group in the copolymer), (116 mg, 0.575 mmol) were dissolved in 1,4-dioxane (2.4 mL). The radical photo initiator, 2-benzyl-2-(dimethylamino)-4′-morpholinobutyrophenone (*Irgacure 369*, 0.84 mg, 2.3 µmol, *20 mol% relative to the polymer’s concentration*) was dissolved separately in the same solvent (0.1 ml) and added to the polymer/thiol solution. The solution was then transferred to a screw-top 8 mL vial, sealed, placed in a UV light box, and irradiated with light (*λ* = 365 nm) while stirring for 1 h. The functionalized polymer was purified by two precipitations into cold hexane to yield the purified product.

**^1^H NMR (400 MHz, CDCl_3_):** *δ(ppm) =* 7.39 – 7.29 (aromatics, m), 5.26 – 5.08 (PLA methine, q, *J* = 7.0 Hz), 5.07 – 4.98 (PLA methine, q, *J* = 7.2 Hz), 4.33 – 4.20 (OC*H*_2_CH_2_N, ddd, *J* = 25.9, 10.6, 4.9 Hz), 4.20 – 4.14 (OC*H*_2_CH_2_S, t, *J* = 6.4 Hz, 1H), 3.66 – 3.47 (NCH_2_C*H*_2_O, m, 4H), 2.62 – 2.35 (C*H*_2_SCH_2_, m, 3H), 1.96 – 1.87 (OCH_2_C*H*_2_CH_2_S, t, *J* = 6.9 Hz), 1.64 – 1.48 (PLA methyl, d, *J* = 7.1 Hz), 1.41 – 1.33 (SCH_2_CH_2_C*H*_2_, t, *J* = 7.4 Hz, 1H), 1.31 – 1.18 (thiol alkyl chain, s), 0.96 – 0.78 (thiol terminal C*H*_2_, t, *J* = 7.0 Hz, 3H).

**^13^C NMR (125 MHz, CDCl_3_):** *δ(ppm) =* 169.94 (PLA carbonyl), 156.14 (N*C*=O), 154.61 (O*C*=OO), 129.49-128.58 (aromatics), 71.79 (benzyl), 69.33 (PLA methine), 67.56-66.38 (O*C*H_2_CH_2_N), 64.97 (O*C*H_2_CH_2_S), 47.73-46.98 (OCH_2_*C*H_2_S), 32.51 (*C*H_2_SCH_2_), 32.25(CH_2_S*C*H_2_), 30.00 (S*C*H_2_), 29.97-28.85 (thiol alkyl chain), 23.03 (thiol penultimate *C*H_2_ unit), 17.18-16.91 (PLA methyl), 14.47 (thiol terminal *C*H_2_ unit).

**SEC (CHCl_3_, RI):** *M*_n_ **=** 11,600 g mol^-1^, *M*_w_ **=** 13,000 g mol^-1^, *Đ*M **=** 1.12

# Analysis and supporting discussion

**Figure S3.** Chemical structure of the screened organocatalysts.

**Table S1.** Organocatalyst screening for the ROCOP of A8NC, P8NC and LLA.

| **Entry** | **Catalyst (mol%)** | **A8NC conv. (%)^a^** | **LLA conv. (%)^a^** | **Time (hours)** | ***M*_n, SEC_ (Da)^b^** | ***Đ*_M_^b^** |
| --- | --- | --- | --- | --- | --- | --- |
| **1** | DPP (10%) | 54 | 0 | 72 | 3.5 | 1.1 |
| **2** | DMAP (10%) | 70 | >95 | 72 | 9.0 | 1.08 |
| **3** | DBU (2%) | >99 | >99 | 0.5 | 10.0 | 1.7 |
| **4^c^** | DPP (10%) / DMAP(20%) | 91 | 95 | 48 | 12.6 | 1.08 |
| **5^d^** | DPP (10%) / DMAP (20%) | 91 | 94 | 48 | 11.2 | 1.15 |

^a^Determined by ^1^H NMR spectroscopy; ^b^Determined by SEC in CHCl_3_, calibrated against PS standards; ^c^[M]_tot_ = 3.0 M, *f*_LA_ = 0.75, *f*_A8NC_ = 0.25, experiments run at 25 °C, initiated by benzyl alcohol. ^d^P8NC was used.

The organocatalysts used for the initial polymerization studies of lactide and the 8-membered cyclic carbonates (A8NC, P8NC) were DBU, DPP and DMAP (Figure S3). The targeted degree of polymerization was 50, the initial total monomer concentration was 3.0 M in CDCl_3,_ the reaction’s temperature was 25 °C and the initiator was benzyl alcohol. The two comonomers were loaded at an equimolar feed ratio (*f*_A8NC_ = *f*_LLA_ = 0.5). Monomer conversion was monitored *via* ^1^H NMR spectroscopy. Small aliquots (50 μL) of the reaction were withdrawn at predetermined time intervals and diluted in C_6_D_6_. The weakly acidic catalyst DPP, in accordance with the literature could not polymerize lactide. After 72 h of reaction time, the carbonate was at 54% conversion, while no polymerization was detected for lactide (Table S1, entry 1). DMAP was able to polymerize both monomers, but with a strong preference towards the cyclic ester. After 72 hours of reaction time, lactide was almost quantitatively converted to polymer, while A8NC was at 72% (Table S1, entry 2). This result indicates a strong difference in the reactivity of the two comonomers, leading to a gradient block-like well-defined copolymer with narrow dispersity indicative of the selectivity of DMAP towards ROP and not transesterification of the open ester.^6^ DBU as a strong base was able to polymerize efficiently and fast both comonomers, leading to full monomer consumption within 30 minutes (Table S1, entry 3). Transesterification side-reactions were dominant and as a direct consequence the dispersity of the polymer was 1.7.

**Figure S4.** ROCOP of A8NC with LLA catalyzed by DPP/DMAP (Table S1, entry 4); Evolution of molar mass distributions *vs* time (*Đ*_M_ values in brackets; in CHCl_3_, calibrated against PS standards). *Aliquot samples were withdrawn during the copolymerization experiment and were analyzed by ^1^H NMR spectroscopy and SEC.*

**Figure S5.** A) Graphical representation of the Kelen - Tüdos^7^ reactivity ratio calculation; B) Graphical representation of the Fineman-Ross reactivity ratio calculation.^8^

The reactivity ratios for LLA/A8NC copolymerizations in CDCl_3_ were determined from the average values obtained for each monomer feed ratio using two of the most popular statistical models in the literature, the Kelen-Tüdos ^7^ and Fineman-Ross (Figure S5).^8^ The average obtained values were revealed to be ***r*_LLA_ = 1.72** and ***r*_A8NC_ = 0.70** from the Kelen-Tüdos model and ***r*_LLA_ = 1.30** and ***r*_A8NC_ = 0.43** from the Fineman-Ross model. In both cases *r*_LLA_ > 1 and *r*_A8NC_ < 1, therefore there seems to be a slight preference for lactide homo-polymerization *vs* cross-polymerization, while the carbonate monomer prefers to react with the other monomer (lactide). This situation would typically result in a random copolymer, as the monomers are not incorporated in a strictly alternating or block fashion, even though relatively small PLA blocky segments cannot be ruled out. Therefore, the repeat unit distribution in P(LLA-*co*-A8NC) is still expected to be approximately random, which is also confirmed by the molar composition values and the NMR spectroscopic data of the purified copolymers.^9, 10^

**Table S2.** ROCOP of A8NC & LLA catalyzed by DPP/DMAP.

| **Entry** | ***f*_LLA_** | **A8NC conv. (%)^a^** | **LLA conv. (%)^a^** | ***F*_A8NC_^a^** | ***F*_LLA_^b^** | ***M*_n, NMR_ (kDa)^a^** | ***M*_n, SEC_**  **(kDa)^c^** | ***Đ*_M_^c^** |
| --- | --- | --- | --- | --- | --- | --- | --- | --- |
| **1** | 0.10 | 92 | 94 | 0.87 | 0.13 | 14.2 | 13.1 | 1.11 |
| **2** | 0.20 | 90 | 93 | 0.77 | 0.23 | 14.2 | 13.5 | 1.1 |
| **3** | 0.30 | 92 | 95 | 0.66 | 0.34 | 14.8 | 13.8 | 1.1 |
| **4** | 0.40 | 92 | 97 | 0.56 | 0.44 | 13.6 | 15.0 | 1.09 |
| **5** | 0.50 | 90 | 94 | 0.48 | 0.52 | 10.4 | 12.6 | 1.08 |
| **6** | 0.60 | 92 | 98 | 0.37 | 0.63 | 8.6 | 12.3 | 1.08 |
| **7** | 0.70 | 92 | 97 | 0.28 | 0.72 | 10.2 | 16.4 | 1.08 |
| **8** | 0.75 | 92 | 95 | 0.24 | 0.76 | 8.2 | 13.3 | 1.13 |
| **9** | 0.80 | 91 | 96 | 0.20 | 0.80 | 9.2 | 15.6 | 1.07 |
| **10** | 0.85 | 91 | 96 | 0.14 | 0.86 | 8.0 | 15.1 | 1.12 |
| **11** | 0.90 | 93 | 96 | 0.09 | 0.91 | 10.0 | 16.7 | 1.06 |

^a^Determined by ^1^H NMR spectroscopy; *f*_LLA_ is the molar ratio of l-LA in the feed; *F*_A8NC_ is the experimental molar incorporation of the carbonate into the final copolymer; ^b^*F*_LLA_ = 1-*F*_A8NC_; ^c^Determined by SEC in CHCl_3_, calibrated against PS standards; Reaction conditions: [M]_tot_ = 3.0 M in CDCl_3_, [M]_0_/[I]_0_/[DPP]_0_/[DMAP]_0_ = 50/1/5/10, 25 °C, time = 48 h.

 **Figure S6.** ^1^H NMR spectrum of a P(LLA-*co*-A8NC_24%_) copolymer (Table S2, entry 8); (*CHCl_3_); (CDCl_3_, 500 MHz).

**Figure S7.** ^1^H NMR spectrum of a P(LLA-*co*-P8NC_26%_) copolymer (*CDCl_3_, **polymer chain-end), (CDCl_3_, 500 MHz).

**Scheme S3.** Synthesis of a P(A8NC)-*block*-PLLA diblock copolymer.

**Figure S8.** ^13^C NMR spectrum of a P(LLA-*co*-P8NC_26%_) copolymer; (CDCl_3_, 500 MHz).

**Figure S9.** ^1^H NMR spectra of a A) P(A8NC)-*block*-PLLA diblock copolymer; B) P(LLA-*co*-A8NC_48%_) statistical copolymer; (CDCl_3_, 500MHz).

As a control experiment, a diblock copolymer P(A8NC)-*block*-PLLA was synthesized (Scheme S3, Figures S9, S10). 30 equivalents of A8NC were first polymerized using DBU as an organocatalyst followed by an *in-situ* addition (with no quenching or purification) of 30 equivalents of lactide until the completion of the polymerization which ultimately yielded the diblock copolymer (*M*_n, SEC_ = 12.2 kDa, *Đ*_M_ = 1.15). ^1^H and ^13^C NMR spectroscopy of this polymer revealed no sequence (ester-carbonate) peaks compared to the statistical analogues. The small methyl resonances at Figure S9 located at *δ* = 1.49 – 1.53 ppm of the ^1^H NMR spectrum can be identified as the final repeating unit of the PLLA block and not a sequence resonance. ^13^C NMR spectroscopy also reveals no sequence peaks (Fig. S10). Specifically looking at the polycarbonate region, the up-field resonance of the ester-carbonate bond is missing. At the polyester region, the carbonyl carbon peak of PLLA is depicted as a well-resolved singlet with no evident neighboring peaks. This result shows a high isotactic enchainment of the PLLA chain with ester units of identical neighboring chemical environment (*i.e.,* no carbonate moieties seem to be present). It is also worth noting that the shape of the polycarbonate peaks in the ^1^H NMR spectrum of the diblock copolymer is much sharper and more defined than in the statistical analogue. This comes as a validation of the previous note that randomizing the ester and carbonate moieties reflects on the appearance and definition of their respective ^1^H NMR spectra.

**Figure S10.** ^13^C NMR spectra focused on the carbonyl region of the polyester and polycarbonate of A) a statistical P(LLA-*co*-A8NC_48%_) copolymer; B) a diblock P(A8NC)-*block*-PLLA copolymer; (CDCl_3_, 125MHz).

**Figure S11.** DOSY NMR spectrum of a P(LLA-*co*-A8NC) copolymer (*CDCl_3_); (CDCl_3_, 500 MHz).

DOSY-NMR spectroscopy was employed to show that the obtained copolymers were diffusing as one entity and not a mixture of homopolymers (Fig. S11). From the obtained spectrum, it is shown that the ester and carbonate units diffuse at the same rate, thus are bonded covalently and are both attached to a benzyl alcohol α-end group.

**Figure S12.** A) MALDI-ToF MS spectrum of a low molar mass P(LLA-*co*-A8NC) copolymer (*f*_A8NC_ = 0.5); B) Zoom-in and further analysis of separate mass peaks; (reflector mode).

Analysis of the obtained poly(ester-carbonate)s was carried out *via* MALDI-ToF MS (Fig. S12). A low molar mass copolymer was obtained by polymerizing LLA and A8NC (total [M]_0_/[I]_0_ = 50, *f*_A8NC_ = 0.5, initiated by benzyl alcohol and catalyzed by DPP/DMAP) and quenching the reaction after 6 h (*M*_n, SEC_ = 6.2 kDa, *Đ*_M_ = 1.07). The analysis of the obtained mass spectrum showed the presence of both monomers in the polymer chain and multiple sodium charged benzyl alcohol α-capped distributions separated by regular spacings of either 144 m/z or 215 m/z which correspond to the molar mass of the monomers. These distributions can be further analyzed as distinct monomer sequences by applying the equation $108.10+23.00+\left( 215.09 \times\boldsymbol{A} \right)+(144.06 \times\boldsymbol{B})$, where ***A*** is the number of carbonate units and ***B*** is the number of lactidyl units, 23.00 is the molar mass of a sodium atom and 108.10 is the molar mass of the initiator. The absence of secondary distribution demonstrates the excellent control of the copolymerization which leads to a well-defined copolymer with high end-group fidelity and no by-product from side-reactions.

**Figure S13.** A representative full DSC thermogram of a P(LLA-*co*-A8NC_9%_) copolymer. Both *T*_g_s can be observed (A, C) as well as both *T*_m_s (B, E) of the two heating cycles. A small cold crystallization peak during the 2^nd^ heating scan (D) can also be identified; (10 °C/min, N_2_ atmosphere).

**Table S3.** Thermal analysis of alkene-functional poly(ester-carbonate)s prepared from the DPP/DMAP organocatalytic ROP of l-lactide, d-lactide and A8NC carbonate monomer.

| **Entry** | ***F*_LLA_^a^** | ***Ł*_LA_^a^** | ***Ł_A8NC_*^b^** | ***X*_c_^c^ (%)** | ***T*_g_^d^ (°C)** | ***T*_m1_^e^ (°C)** | **Δ*H*_m1_^f^ (J/g)** | ***T*_m2_^g^ (°C)** | **Δ*H*_m2_^h^ (J/g)** |
| --- | --- | --- | --- | --- | --- | --- | --- | --- | --- |
| **1** | 0 | - | - | - | -21.5 | - | - | - | - |
| **2** | 0.13 | 2.14 | 9.33 | - | -8.0 | - | - | - | - |
| **3** | 0.23 | 2.43 | 5.41 | - | -4.9 | - | - | - | - |
| **4** | 0.34 | 3.82 | 3.34 | - | 4.7 | - | - | - | - |
| **5** | 0.44 | 4.56 | 2.65 | - | 7.1 | - | - | - | - |
| **6** | 0.52 | 5.81 | 2.15 | - | 13.6 | - | - | - | - |
| **7** | 0.63 | 7.8 | 1.78 | - | 19.2 | - | - | - | - |
| **8** | 0.72 | 9.5 | 1.48 | - | 25.7 | - | - | - | - |
| **9** | 0.76 | 10.9 | 1.38 | 8.3 | 30.6 | 94.44 | 7.735 | - | - |
| **10** | 0.8 | 12.5 | 1.32 | 19.0 | 34.6 | 103.5 | 17.69 | - | - |
| **11** | 0.86 | 13.8 | 1.16 | 29.5 | 37.6 | 121.94 | 27.44 | 125.36 | 8.62 |
| **12** | 0.91 | 15.2 | 1.05 | 34.2 | 44.6 | 133.57 | 31.86 | 135.44 | 25.58 |
| **13** | 1 | - | - | 54.2 | 50.6 | 151.44 | 50.44 | 150.68 | 45.42 |
| **14^i^** | 1 |  |  | 52.7 | 49.8 | 152.76 | 49.04 | 155.39 | 48.41 |

^a^Determined *via* ^1^H NMR spectroscopy using Eq. S1; *Ł*_LA_ calculated based on Eq. S3; ^b^Determined *via* ^13^C NMR spectroscopy; *Ł*_A8NC_ calculated based on Eq. S2; ^c^Degree of crystallinity of PLA blocks: *X*_c_ = Δ*H*_m1_/Δ*H^0^*_m_, Δ*H^0^*_m_ theoretical maximum enthalpy of fusion value for polylactide crystallites of 100% *X*_c_ = 93.6 J/g;^11, 12^ ^d^Determined from the 2^nd^ heating scans; ^e^Melting temperature measured by DSC analysis in the first heating run; ^f^Melting enthalpy in the 1^st^ heating run; ^g^Melting temperature obtained in the second heating run from samples in the melt; ^h^Melting enthalpy in the 2^nd^ heating run; ^i^PDLA; DSC conditions: 10 °C/min for all cycles, under N_2_ gas.

The molar composition of the copolymers was determined by ^1^H NMR spectroscopy by comparing the integral value of the polycarbonate’s pendant -C*H_2_*-CH=CH_2_ two protons against the integral value of the overlapped resonances of the polylactide methine protons with the polycarbonate allyl CH_2_-CH=C*H_2_* two protons, following equation (S1):

$F_{A-8NC}=\frac{I_{g}}{I_{i}+I_{c}+I_{LC}}$ (Eq. S1)

**Figure S14.** A) Chart of *T*_m1_ vs *F*_LLA;_ B) Chart of degree of crystallinity *X*_c_ (%) vs *Ł*_LA._


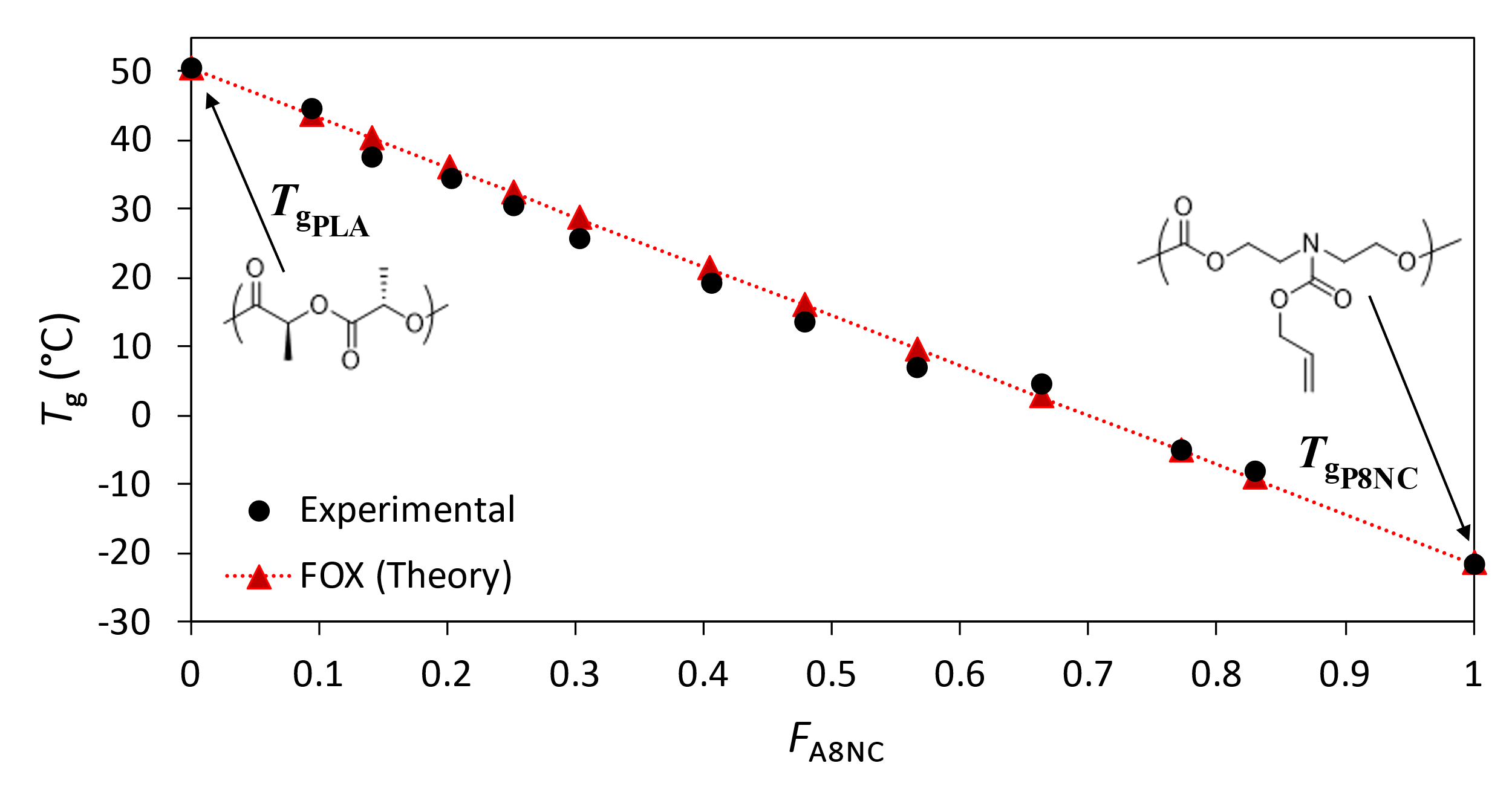


**Figure S15.** Plot of theoretical *T*_g_ values of the obtained copolymers calculated from the Fox equation and the experimental values *versus* the molar fraction of the carbonate unit in the copolymer chain (*T*_g_ values are from the 2^nd^ heating scans).

The Fox equation^15-17^ (Eq. S2) can be used to predict the *T*_g_ of copolymers consisting of any ratio of two monomers if the *T*_g_s of the homopolymers are known and a statistical structure of the copolymer is obtained:

$\frac{1}{T_{g}}=F_{A8NC}\left( \frac{1}{T_{g_{PA8NC}}}-\frac{1}{T_{g_{PLA}}} \right)+\frac{1}{T_{g_{PLA}}}$ (Eq. S2)

Where *F*_A8NC_ is the molar fraction of monomer A8NC in the copolymer and *T*_gLA_ and *T*_gA8NC_ represent the glass transition temperatures of the PLLA and P(A8NC) homopolymers, respectively. P(A8NC) homopolymer is an amorphous polymer independently of its molar mass, with a *T*_g_ = -21.47 °C.^18^

----------------------------------------------------------------------------------------------------------------------------------

As a control experiment, the DSC of the diblock copolymer P(A8NC)_30_-*block*-PLLA_30_ was recorded (Figure S16). In a diblock copolymer, phase separation is expected since the two blocks of the material are immiscible in the melt, so two distinct *T*_g_s are usually observed. On the first heating run the *T*_g_ of the polycarbonate block can easily be identified at 0 °C and is the predominant one. A second much more dwarfed *T*_g_ around 51 °C can also be seen which is attributed to the PLLA block. A melting temperature at 109.2 °C was also observed, therefore the PLLA block was long enough in order to crystallize. No crystallization or melting events were recorded during the 2^nd^ heating run and a single *T*_g_ could now be detected at 6.67 °C. This could be a result of melt-blending and shift of the two distinct *T*_g_s towards a single value.^19^

**Figure S16.** DSC thermogram (1^st^ run) of a diblock P(A8NC)_30_-*block*-PLLA_30_. The P(A8NC) *T*_g_ (A), the PLLA *T*_g_ (B) and the PLLA *T*_m_ (C) can be observed. (10 °C/min, N_2_ atmosphere).

**Table S4.** ROCOP of l-LA and P8NC catalyzed by DPP/DMAP.

| **Entry** | ***f*_LLA_** | **P8NC conv. (%)^a^** | **LLA conv. (%)^a^** | ***F*_P8NC_^a^** | ***F*_LLA_^b^** | ***M*_n, NMR_ (kDa)^a^** | ***M*_n, SEC_**  **(kDa)^c^** | ***Đ*_M_^c^** |
| --- | --- | --- | --- | --- | --- | --- | --- | --- |
| **1** | 0.75 | 94 | 91 | 0.26 | 0.74 | 8.1 | 11.2 | 1.15 |
| **2** | 0.80 | 95 | 93 | 0.19 | 0.81 | 6.7 | 10.7 | 1.09 |
| **3** | 0.85 | 93 | 92 | 0.14 | 0.86 | 6.3 | 11.2 | 1.07 |
| **4** | 0.90 | 97 | 92 | 0.08 | 0.92 | 8.6 | 12.6 | 1.09 |

^a^Determined by ^1^H NMR spectroscopy; ^b^*F*_LLA_ = 1-*F*_P8NC_; ^c^Determined by SEC in CHCl_3_, calibrated against PS standards; Reaction conditions: [M]_tot_ = 3.0 M in CDCl_3_, [M]_0_/[I]_0_/[DPP]_0_/[DMAP]_0_ = 50/1/5/10, 25 °C, time = 48 h.

**Table S6.** Thermal analysis of P(LLA-*co*-P8NC) copolymers.

| **Entry** | ***F*_LLA_^a^** | ***Ł*_LA_^a^** | ***Ł_P8NC_*^b^** | ***X*_c_^c^ (%)** | ***T*_g_^d^ (°C)** | ***T*_m1_^e^ (°C)** | **Δ*H*_m1_^f^ (J/g)** | ***T*_m2_^g^ (°C)** | **Δ*H*_m2_^h^ (J/g)** |
| --- | --- | --- | --- | --- | --- | --- | --- | --- | --- |
| **1** | 0.74 | 8.84 | 1.45 | - | 37.53 | - | - | - | - |
| **2** | 0.81 | 10.65 | 1.36 | 17.8 | 41.24 | 95.92 | 16.64 | - | - |
| **3** | 0.86 | 12.07 | 1.38 | 28.6 | 42.9 | 118.07 | 26.62 | - | - |
| **4** | 0.92 | 23.83 | 1.28 | 26.7 | 45.6 | 130.24 | 24.87 | - | - |
| **5** | 1 | - | - | 54.24 | 50.62 | 151.44 | 50.44 | 150.68 | 45.42 |

^a^Determined *via* ^1^H NMR spectroscopy; ^b^Determined *via* ^13^C NMR spectroscopy; ^c^Degree of crystallinity of PLA blocks: *X*_c_ = Δ*H*_m1_/Δ*H^0^*_m_, Δ*H^0^*_m_ theoretical maximum enthalpy of fusion value for polylactide crystallites of 100% *X*_c_ = 93.6 J/g;^11, 12^ ^d^Determined from the 2^nd^ heating scans; ^e^Melting temperature measured by DSC analysis in the first heating run; ^f^Melting enthalpy in the 1^st^ heating run; ^g^Melting temperature obtained in the second heating run from samples in the melt; ^h^Melting enthalpy in the 2^nd^ heating run; DSC conditions: 10 °C/min for all cycles, N_2_ atmosphere.

**Figure S17.** Experimental *T*_g_ values of all P(LLA-*co*-P8NC) copolymers (black dots) against the theoretical model (red dotted line) derived from the Fox equation.

The calculation of the l-lactoyl (*Ł*_LA_) and carbonate (*Ł_A8NC_*) average block sequence lengths of all synthesized copolymers (Table S3) were calculated from their respective ^1^H and ^13^C NMR spectra and they were based on modified previously published methods (equations S3, S4).^13, 14^ Obviously, the trend of increasing block lengths follows the reactivity ratios of the two comonomers as well as their molar loading in the polymerization feed. Therefore, the plot of *Ł*_LA,_ *Ł_A8NC_* *vs* the *f*_LLA_ shows a gradual alteration of the block length values, which is not linear since the copolymerization is not an ideal 100% random or alternating (Figure S18).

$L_{A8NC}=\frac{I_{CC}}{I_{CL}}+1$ (Eq. S3)

$L_{LA}=\frac{{(I}_{i}+I_{c})-I_{g}}{I_{LC}}+1$ (Eq. S4)

**Figure S18.** Graphical depiction of the evolution of lactoyl and carbonate average block length values *vs* the molar feed of lactide. The gradient blue colored area covers the semi-crystalline region of the copolymers.

**Figure S19.** A) TGA thermograms (thermal decomposition profiles); B) 1^st^ derivative of % weight *vs* temperature (DTG) of pure PLLA and P(LLA-*co*-A8NC) copolymers of varying carbonate incorporation.

**Figure S20.** TGA thermogram and 1^st^ derivative of % weight *vs* temperature of a P(LLA-*co*-P8NC_14%_) copolymer; (10 °C/min, N_2_ atmosphere).

**Table S6.** Thermal characterizations observed after stereocomplexation of P(LLA-*co*-A8NC) copolymers with PDLA and with copolymers of identical composition and opposite chirality, P(DLA-*co*-A8NC) (equimolar quantities).

| **Entry** | **Polymer** | ***T*_g_^a^ (°C)** | ***T*_m1_^b^ (°C)** | **Δ*H*_m1_^c^ (J/g)** | ***T*_m2_^d^ (°C)** | **Δ*H*_m2_^e^ (J/g)** | ***T*_c_^f^ (°C)** |
| --- | --- | --- | --- | --- | --- | --- | --- |
| **1** | P(LLA-*co*-A8NC_9.4%_) | 48.4 | 193.7 | 39.2 | 193.2 | 39.2 | 98.7 |
| **2** | P(LLA-*co*-A8NC_14.1%_) | 45.8 | 186.9 | 30.6 | 181.2 | 24.9 | 114.0 |
| **3** | P(LLA-*co*-A8NC_20.2%_) | 45.9 | 180.0 | 18.9 | 177.5 | 14.0 | 119.6 |
| **4** | P(LLA-*co*-A8NC_24.7%_)^g^ | 39.8 | 171.6 | 19.6 | 170.7 | 0.3 | 93.4^h^ |
| **5** | P(LLA-*co*-A8NC_9.4%_) | 34.9 | 194.1 | 45.6 | 192.9 | 38.8 | 122.3^i^ |
| **6** | P(LLA-*co*-A8NC_14.1%_) | 31.4 | 179.7 | 42.0 | 178.8 | 32.1 | 92.4 |
| **7** | P(LLA-*co*-A8NC_20.2%_) | 23.9 | 167.5 | 23.2 | 170.6 | 18.5 | 103.3 |
| **8** | P(LLA-*co*-A8NC_24.7%_)^g^ | 26.5 | 155.8 | 21.3 | 156.1 | 13.6 | 102.6^h^ |

All samples annealed at 150 °C *in vacuo* for 24h before analysis; Entries 1-4 describe equimolar blends with PDLA; Entries 5-8 describe equimolar blends with copolymers of identical composition and opposite chirality, P(DLA-*co*-A8NC); **DSC conditions**: 5mg samples, N_2_ atmosphere, 10 °C min^-1^ heating and cooling rates, non-annealed samples; ^a^*T*_g_ at the 2^nd^ heating scan; ^b^Melting temperature measured by DSC analysis in the first heating run; ^c^Samples obtained by solution crystallization; ^d^Melting temperature measured by DSC analysis in the second heating run; ^e^Results obtained from samples in the melt; ^f^Crystallization temperature obtained in the second heating run from samples in the melt; ^g^5 °C min^-1^ heating and cooling rates; ^h^Crystallization temperature obtained in the 1^st^ heating run; ^i^Crystallization peak at the cooling run.


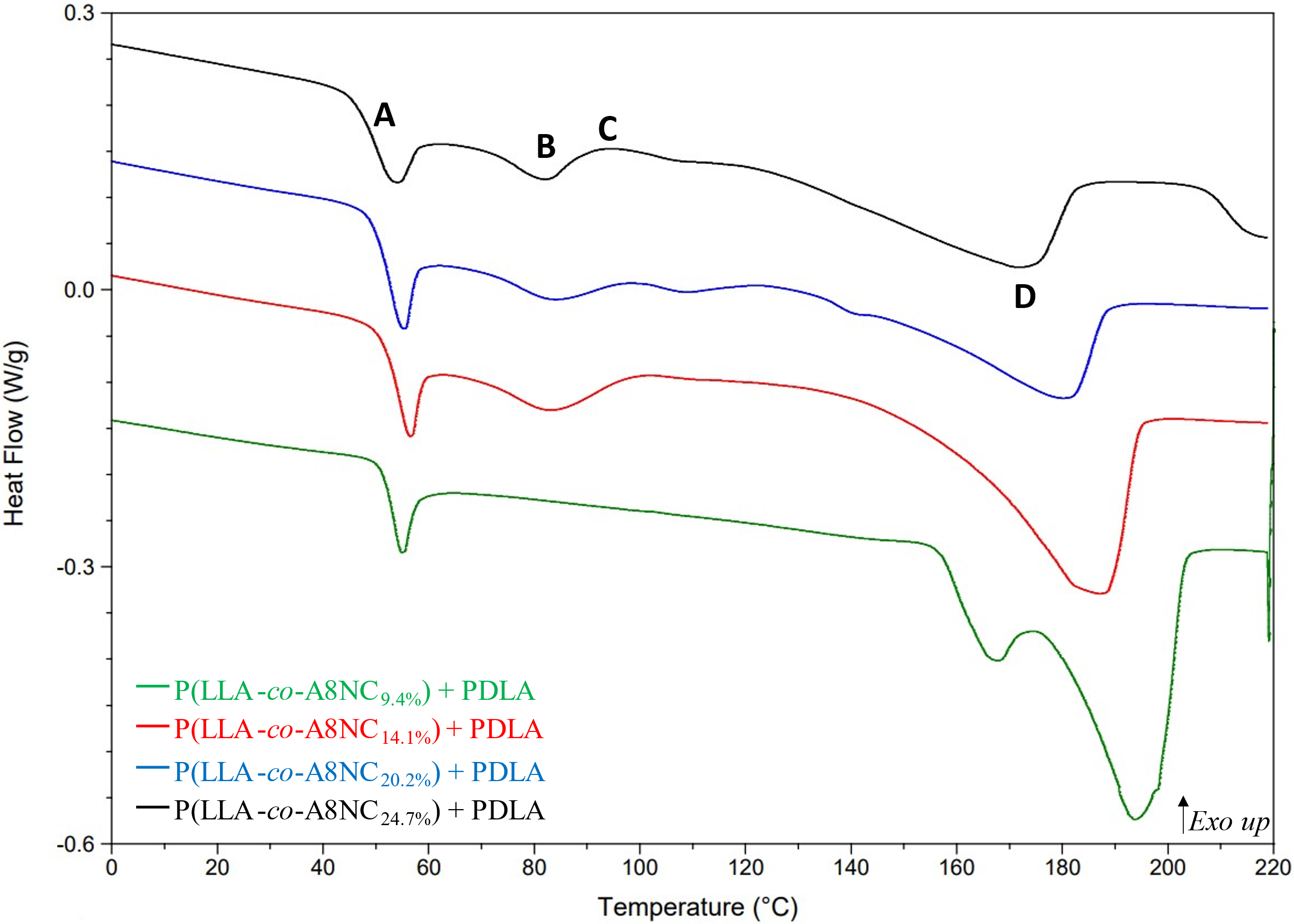


**Figure S21.** Stacked DSC thermograms of equimolar stereocomplexes of P(LLA-*co*-A8NC) copolymers with PDLA (Table S6, entries 1-4, post-annealing 1^st^ heating scans). The thermograms display the glass transition (A), homochiral melting (B), crystallization peak of the stereocomplexes, (C) and the melting temperature of the resulting SCs (D); (N_2_ atmosphere, heating/cooling rate of 10 °C min^-1^ unless stated otherwise).


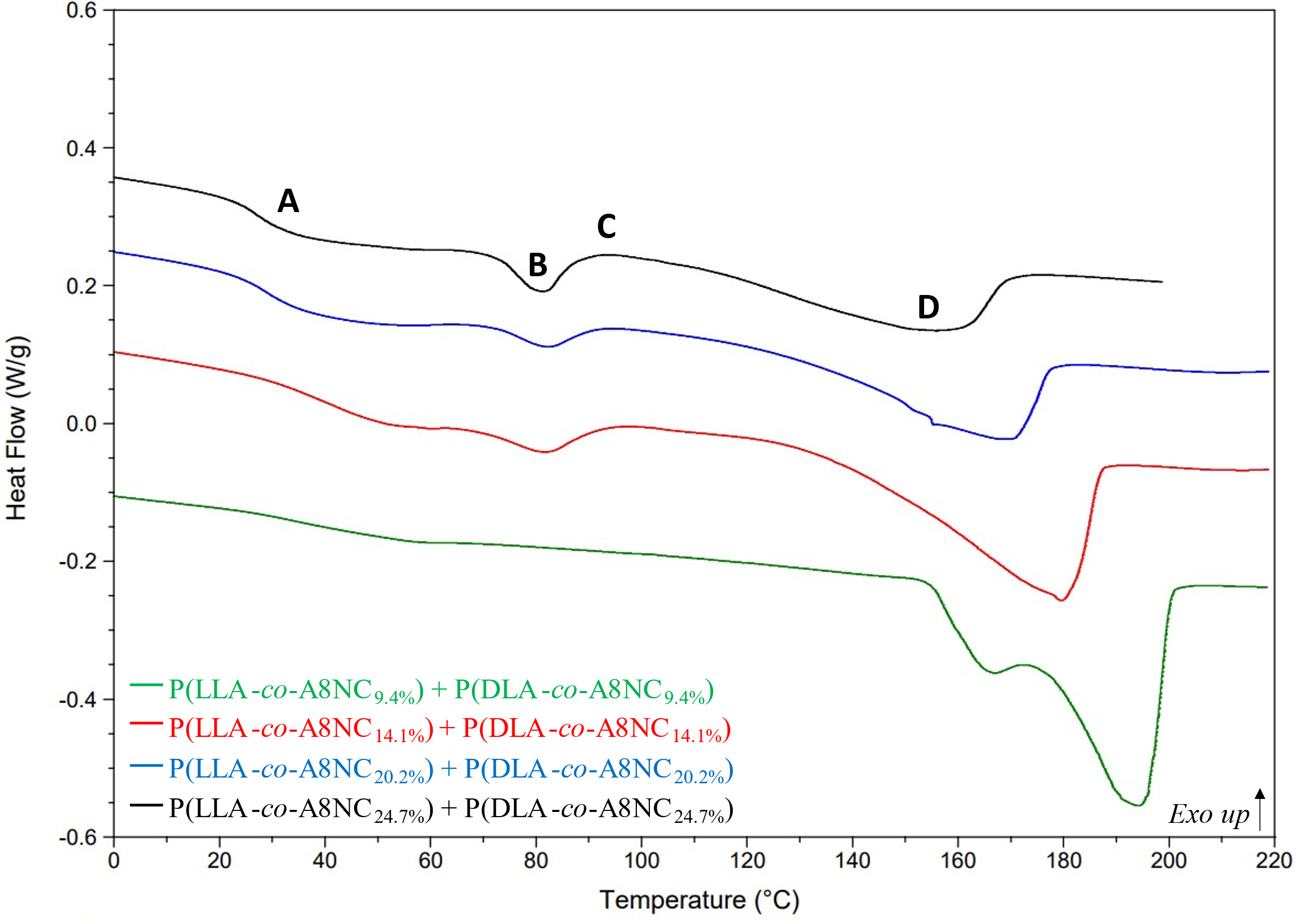


**Figure S22.** Stacked DSC thermograms of equimolar stereocomplexes of P(LLA-*co*-A8NC) copolymers with P(DLA-*co*-A8NC) copolymers of identical composition, post-annealing (Table S6, entries 5-8, 1^st^ heating scans). The thermograms display the glass transition (A), homochiral melting (B), crystallization peak of the stereocomplexes, (C) and the melting temperature of the resulting SCs (D); (N_2_ atmosphere, heating/cooling rate of 10 °C min^-1^ unless stated otherwise).

**Figure S23.** TGA thermograms of the equimolar stereocomplexes of [P(LLA-*co*-A8NC) + PDLA] blends (Table S6, entries 1-4) compared to a pure PLA-SC; (N_2_ atmosphere, heating rate of 10 °C min^-1^).

The TGA thermograms of the [P(LLA-*co*-A8NC) + PDLA] blends when plotted against the [PLLA+PDLA] blend as reference show that the increase of the carbonate’s incorporation to the copolymer has a detrimental effect on the thermal stability of the blends (Figure S23). Even at 9.4 mol% of carbonate units in the copolymer, the thermal degradation profile of the blend is distinctly different to the pure PLA stereocomplex, and as the incorporation of the polycarbonate further increases, the blends become more thermally labile, with the *T*_d_ gradually decreasing from 265 to 225 °C. Compared to the non-stereocomplexed P(LLA-*co*-A8NC) copolymers (Figure S19), there is a slight increase in the *T*_d_ ranging from 10 – 25 °C. The 1^st^ derivative of the thermal degradation curves expressed in % mass loss *vs* temperature clearly show a mix of major thermal degradation profiles which proportionally shift towards a lower *T*_d_, *ca.* 375 °C for the [P(LLA-*co*-A8NC_9.4%_) + PDLA] blend to 265 °C for the [P(LLA-*co*-A8NC_24.7%_) + PDLA] blend, as the carbonate content of the copolymer increases (Fig. S24).

**Figure S24.** 1^st^ derivative of % weight *vs* temperature (DTG) of equimolar stereocomplexes of [P(LLA-*co*-A8NC) + PDLA] blends (Table S6, entries 1-4) compared to a pure PLA-SC; (N_2_ atmosphere, heating rate of 10 °C min^-1^).

**Figure S25.** TGA thermograms of the equimolar stereocomplexes of [P(LLA-*co*-A8NC) + P(DLA-*co*-A8NC)] blends (Table S6, entries 5-8) compared to a pure PLA-SC; (N_2_ atmosphere, heating rate of 10 °C min^-1^).

The TGA thermograms of the [P(LLA-*co*-A8NC) + P(DLA-*co*-A8NC)] blends when plotted against the [PLLA+PDLA] blend as reference show that the increase of the carbonate’s incorporation to the copolymer has a detrimental effect on the thermal stability of the blends as previously observed (Figure S25). The deviation from the pure PLA stereocomplex thermal degradation profile is more evident in these blends as no pure PLA segment is used. As the incorporation of the polycarbonate further increases, the materials become more thermally labile, with the *T*_d_ gradually decreasing from *ca.* 350 to 230 °C. Again, as previously described, when compared to their non-stereocomplexed analogues, these materials exhibit an enhanced thermal stability ranging from ∼ 10 °C to 25 °C. The 1^st^ derivative of the thermal degradation curves (Figure S26) clearly displays the drift of the thermal stability towards lower temperature values, with the ester-rich structures retaining their thermostability versus the carbonate-rich materials.


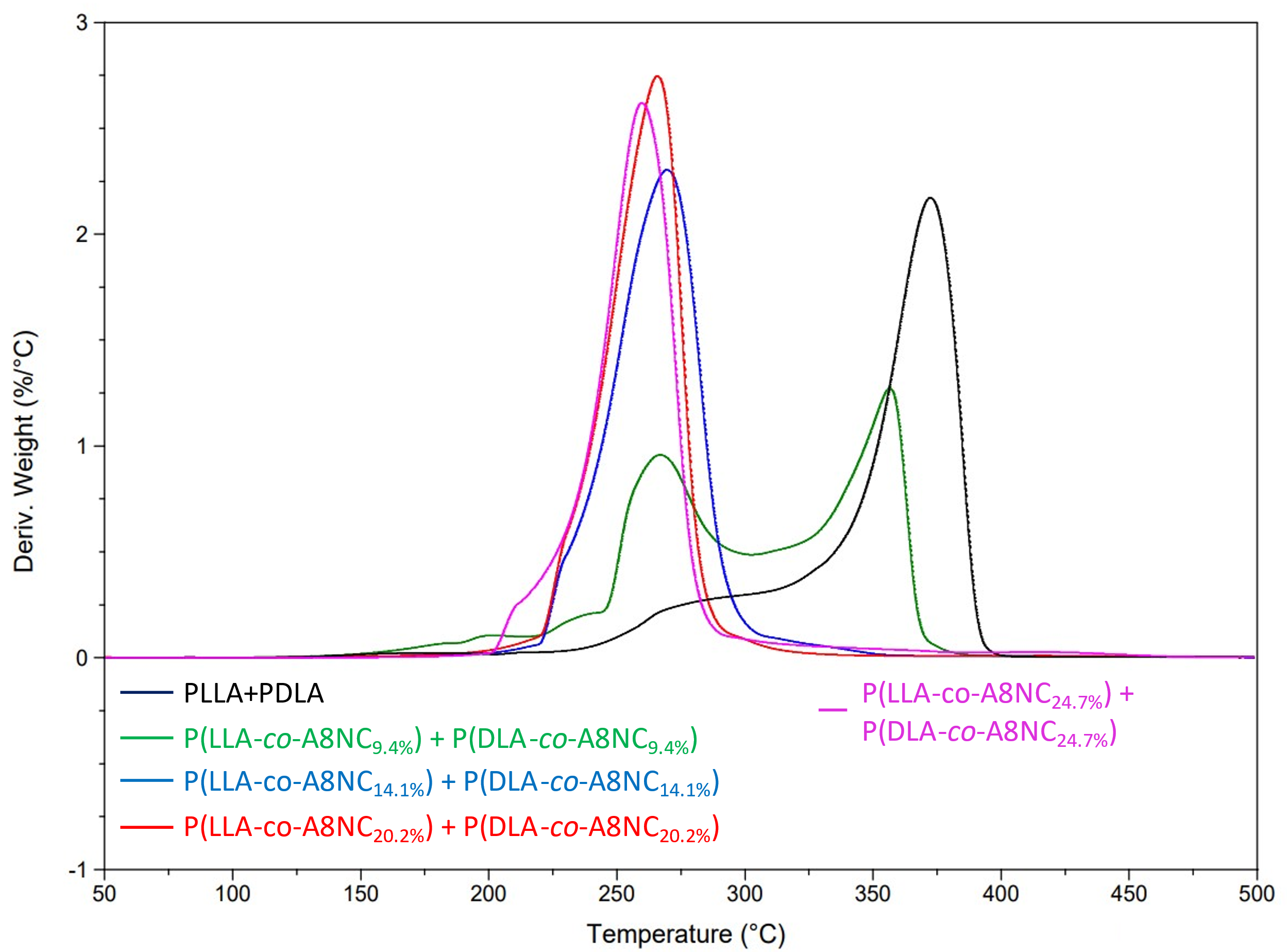


**Figure S26.** 1^st^ derivative of % weight *vs* temperature (DTG) of equimolar stereocomplexes of [P(LLA-*co*-A8NC) + P(DLA-*co*-A8NC)] blends (Table S6, entries 5-8) compared to a pure PLA-SC; (N_2_ atmosphere, heating rate of 10 °C min^-1^).

**Figure S27.** Stacked DSC thermograms of P(LLA-*co*-A8NC_9.4%_) : PDLA blends (post-annealing, 1^st^ heating scans). The thermograms display the glass transition (A), crystallization peak (B), homochiral melting (C), and the melting temperature of the resulting SCs (D); (N_2_ atmosphere, heating/cooling rate of 10 °C min^-1^ unless stated otherwise).

**Figure S28.** Stacked DSC thermograms of P(LLA-*co*-A8NC_9.4%_) : PDLA blends (post-annealing, 2^nd^ heating scans). The thermograms display the glass transition (A), crystallization peak (B) and the melting temperature of the resulting SCs (C); (N_2_ atmosphere, heating/cooling rate of 10 °C min^-1^ unless stated otherwise).

**Figure S29.** Stacked DSC thermograms of P(LLA-*co*-A8NC_9.4%_) : P(DLA-*co*-A8NC_9.4%_) blends (post-annealing, 1^st^ heating scans). (N_2_ atmosphere, heating/cooling rate of 10 °C min^-1^ unless stated otherwise).

**Figure S30.** Stacked DSC thermograms of P(LLA-*co*-A8NC_9.4%_) : P(DLA-*co*-A8NC_9.4%_) blends (post-annealing, 2^nd^ heating scans). (N_2_ atmosphere, heating/cooling rate of 10 °C min^-1^ unless stated otherwise).

**Figure S31.** DSC thermograms (1^st^ heating scans) of a [PLLA-*block*-P(A8NC) + PDLA] equimolar blend annealed at A) 120 °C and B) 150 °C; (N_2_ atmosphere, heating/cooling rate of 10 °C min^-1^).

The melting enthalpies of the two events were of similar magnitude (*ca.* 35 J/g), showing the presence of the two crystalline domains in the material at an equilibrium. The 2^nd^ heating scan showed no melting event, constituting the material completely amorphous. To eliminate the PDLA homochiral melting, a further annealing at 150 °C was done. Subsequently, the DSC 1^st^ heating scan revealed only one endotherm at *ca.* 193 °C identifying to the stereocomplex melting. This value was 7 °C higher than the previous SC *T*_m_ because of the disruption of the homochiral crystals which now contributed to the enhancement of the crystallinity of the stereocomplexed domain. The 2^nd^ heating scan revealed no crystallization nor melting events once again, so the material after its melting process is unable to reform into a crystal, possibly due to emphasized presence of the carbonate which hinders the proper alignment of the PLLA and PDLA segments in order to stereocomplex.

**Table S7.** Thermal characterizations observed after stereocomplexation of P(LLA-*co*-P8NC) copolymers with PDLA and with copolymers of identical composition and opposite chirality (equimolar quantities).

| **Entry** | **Polymer** | ***T*_g_^a^ (°C)** | ***T*_m1_^b^ (°C)** | **Δ*H*_m1_^c^ (J/g)** | ***T*_m2_^d^ (°C)** | **Δ*H*_m2_^e^ (J/g)** | ***T*_c_^f^ (°C)** |
| --- | --- | --- | --- | --- | --- | --- | --- |
| **1** | P(LLA-*co*-P8NC_10.7%_) | 47.52 | 185.45 | 33.63 | 178.38 | 14.67 | 134.71 |
| **2** | P(LLA-*co*-P8NC_15.2%_) | 47.38 | 176.73 | 27.9 | 179.06 | 28.1 | 124.46 |
| **3** | P(LLA-*co*-P8NC_19.8%_) | 42.72 | 166.4 | 11.76 | 164.4 | 10.91 | 104.78 |
| **4** | P(LLA-*co*-P8NC_10.7%_) | 44.53 | 181.08 | 43.38 | 179.01 | 37.95 | 111.23 |
| **5** | P(LLA-*co*-P8NC_15.2%_) | 39.17 | 172.87 | 29.79 | 174.56 | 7.07 | 132.6 |
| **6** | P(LLA-*co*-P8NC_19.8%_) | 44.7 | 158.4 | 5.81 | 162.4 | 4.09 | 117.6 |

All samples annealed at 150 °C *in vacuo* for 24h before analysis; Entries 1-3 describe equimolar blends with PDLA; Entries 4-6 describe equimolar blends with copolymers of identical composition and opposite chirality, P(DLA-*co*-P8NC); DSC conditions: 5mg samples, N_2_ atmosphere, 10 °C/min heating and cooling rates, non-annealed samples; ^a^*T*_g_ at the 2^nd^ heating scan; ^b^Melting temperature measured by DSC analysis in the first heating run; ^c^Samples obtained by solution crystallization; ^d^Melting temperature measured by DSC analysis in the second heating run; ^e^Results obtained from samples in the melt; ^f^Crystallization temperature obtained in the second heating run from samples in the melt.

**Figure S32.** Stacked DSC thermograms of equimolar stereocomplexes of P(LLA-*co*-P8NC) copolymers with PDLA (Table S7, entries 1-3, 1^st^ heating scans). The thermograms display the glass transition (A), crystallization peak of the stereocomplexes (B), and the melting temperature of the resulting SCs (C); (N_2_ atmosphere, heating/cooling rate of 10 °C min^-1^).

**Figure S33.** Stacked DSC thermograms of equimolar stereocomplexes of P(LLA-*co*-P8NC) copolymers with P(DLA-*co*-P8NC) of identical composition (Table S7, entries 4-6, 2^nd^ heating scans). The thermograms display the glass transition (A), crystallisation peak of the stereocomplexes (B) and the melting temperature of the resulting SCs (C); (N_2_ atmosphere, heating/cooling rate of 10 °C min^-1^).

**Figure S34.** Plots of *T*_m1_ vs *f*_P8NC_ (%) (1^st^ heating scans) of the equimolar stereocomplexes comprised of A) [P(LLA-*co*-P8NC) + PDLA] (Table S7, entries 1-3); B) [P(LLA-*co*-P8NC) + P(DLA-*co*-P8NC)] (Table S7, 4-6).

**Figure S35.** TGA (A) and DTG (B) thermograms of [P(LLA-*co*-P8NC_10.7%_) + PDLA] and [P(LLA-*co*-P8NC_10.7%_) + P(DLA-*co*-P8NC_10.7%_)] blends compared to the neat [PLLA+PDLA] stereocomplex showcasing the comparison in the thermal degradation profile of these materials; (N_2_ atmosphere, heating rate of 10 °C min^-1^).


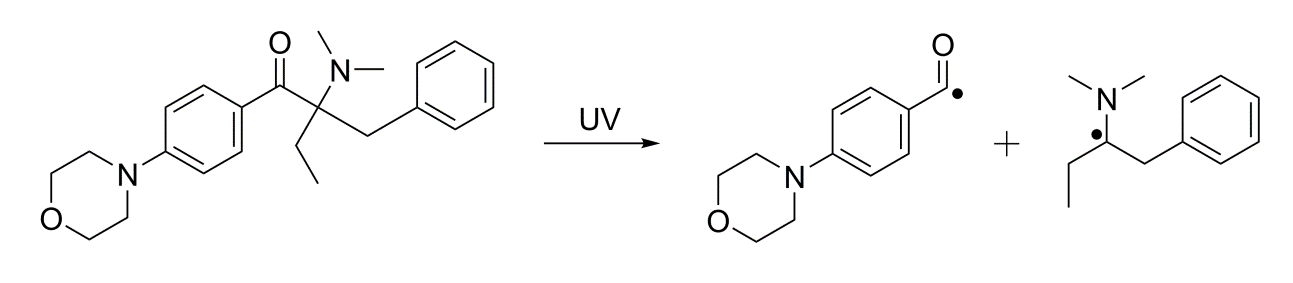


**Scheme S4.** Structure and UV light activation of the Irgacure^®^ 369 photo initiator used for the thiol-ene reactions in this work.

The photo initiator 2-benzyl-2-(dimethylamino)-4'-morpholinobutyrophenone (Irgacure^®^ 369, Scheme S4) was chosen due to its commercial availability and broad absorption range. Furthermore, it has been successfully applied to the functionalization of similar type polymers.^20^

**Table S8.** Thiol functionalized polymers showing thiolene reaction conversion (%), molar mass, and dispersity *Đ_M_* of the resulting thiol-functional copolymers.

| **Entry** | **Copolymer** | **Thiol (equivalents used)^a^** | **^b^Conv. (%)** | **^c^M_n_ (kDa) ^d^(Đ_M_)** |
| --- | --- | --- | --- | --- |
| 1 | P(DLA-*co*-A8NC_14.1%_) | 2-Mercaptoethanol (5) | > 99 | 13.2 (1.09) |
| 2 | P(DLA-*co*-A8NC_14.1%_) | 3-Mercaptopropionic acid (5) | > 99 | 14.1 (1.1) |
| 3 | P(DLA-*co*-A8NC_20.2%_) | 1-Hexanethiol (10) | > 99 | 13.0 (1.18) |
| 4 | P(DLA-*co*-A8NC_20.2%_) | Benzyl mercaptan (10) | > 99 | 9.2 (1.16) |
| 5 | P(DLA-*co*-A8NC_25.1%_) | 1-Thioglycerol (10) | > 99 | 10.9 (1.12) |
| 6 | P(LLA-*co*-A8NC_9.4%_) | 1-Dodecanethiol (5) | > 99 | 13.7 (1.14) |
| 7 | P(LLA-*co*-A8NC_14.1%_) | 1-Dodecanethiol (10) | > 99 | 11.6 (1.12) |
| 8 | P(DLA-*co*-A8NC_20.2%_) | 1-Dodecanethiol (10) | > 99 | 12.0 (1.19) |
| 9 | P(DLA-*co*-A8NC_25.1%_) | 1-Dodecanethiol (10) | > 99 | 13.0 (1.21) |

^a^in relation to the alkene functionality of the copolymer; ^b^measured by ^1^H NMR spectroscopy; ^b/c^determined by SEC in CHCl_3_.

**Figure S36.** Stacked ^1^H NMR spectra of a P(LA-*co*-A8NC) copolymers after photo-initiated radical addition of monofunctional thiols; A) Table S8, entry 4, B) Table S8 entry 5, C) Table S8 entry 3, D) Table S8 entry 2, E) Table S8 entry 1; (400MHz, CDCl_3_); (*CDCl_3,_ **ester-carbonate sequence PLA protons (methine & methyl region), *** residual hexane, **** residual DMAP).

**Figure S37.** Size exclusion chromatograms of a P(LLA-*co*-A8NC_14.1%_) copolymer before and after its functionalization with 2-mercaptoethanol (in CHCl_3_, calibrated against PS standards).

**Figure S38.** Stacked FTIR spectra of A) a parent P(LLA-*co*-A8NC_9.4%_) copolymer with the double bond alkene absorption annotated; B) a P(LLA-*co*-A8NC_9.4%_) after photo-initiated radical addition of 1-dodecanethiol, showing the disappearance of the alkene signal and the appearance of the thiol stretch, thereby proving the quantitative replacement of the alkene functionality from the thiol group.

**Figure S39.** WAXD profiles of solution-quenched polymer blends. Both materials were recovered and analyzed directly after precipitation, filtering and drying (no annealing was done). The blue line depicts a stereocomplex formed by an equimolar blend of a P(LLA-*co*-A8NC_15.2%_) and PDLA. The red line depicts a stereocomplex formed by an equimolar blend of a P(DLA-*co*-A8NC_19.8%_) post-functionalized with 1-dodecanethiol and PLLA.

**Table S9.** DSC analysis of thiol-functionalized P(LA-*co*-A8NC) copolymers.

| **Entry** | **Copolymer** | **Thiol functionality** | ***T*_g_ (°C)^a^** | ***T*_m1_ (°C)^b^** | **Δ*H*_m1_ (J/g)^c^** | ***T*_m2_ (°C)^d^** | **Δ*H*_m2_ (J/g)^e^** |
| --- | --- | --- | --- | --- | --- | --- | --- |
| **1** | P(DLA-*co*-A8NC_14.1%_) | 2-Mercaptoethanol | 34.21 | 118.7 | 24.38 | - | - |
| **2** | P(DLA-*co*-A8NC_14.1%_) | 3-Mercaptopropionic acid | 36.94 | 120.04 | 22.4 | - | - |
| **3** | P(DLA-*co*-A8NC_20.2%_) | 1-Hexanethiol | 21.1 | 89.7 | 14.49 | - | - |
| **4** | P(DLA-*co*-A8NC_20.2%_) | Benzyl mercaptan | 52.8 | 104.25 | 11.62 | - | - |
| **5** | P(DLA-*co*-A8NC_25.1%_) | 1-Thioglycerol | 30.41 | - | - | - | - |
| **6** | P(LLA-*co*-A8NC_9.4%_) | 1-Dodecanethiol | 29.24 | 129.13 | 25.97 | 132.01 | 9.36 |
| **7** | P(LLA-*co*-A8NC_14.1%_) | 1-Dodecanethiol | 27.86 | 128.4 | 23.55 | 132.09 | 5.8 |
| **8** | P(DLA-*co*-A8NC_20.2%_) | 1-Dodecanethiol | 41.24 | 95.92 | 16.62 | - | - |
| **9** | P(DLA-*co*-A8NC_25.1%_) | 1-Dodecanethiol | 31.27 | 86.42 | 9.68 | - | - |

**DSC analysis conditions**: 5 mg samples, N_2_ atmosphere, 10 °C/min heating and cooling rates; ^a^*T*_g_ at the 2^nd^ heating scan; ^b^Melting temperature measured by DSC analysis in the first heating run; ^c^Samples obtained by solution crystallization; ^d^Melting temperature measured by DSC analysis in the second heating run; ^e^Results obtained from samples in the melt.

**Table S10.** Thermal characterization observed after stereocomplexation of thiol-functionalized P(LA-*co*-A8NC) type copolymers (*copolymer entries from Table S9*).

| **Entry** | **Copolymer used** | ***T*_g_^a^ (°C)** | ***T*_m1_^b^ (°C)** | **Δ*H*_m1_^c^ (J/g)** | ***T*_m2_^d^ (°C)** | **Δ*H*_m2_^e^ (J/g)** | ***T*_c_^f^ (°C)** |
| --- | --- | --- | --- | --- | --- | --- | --- |
| **I** | **(6)** | 46.58 | 185.53 | 33.76 | 188.2 | 31.34 | 90.83 |
| **II** | **(7)** | 46.58 | 179.6 | 16.88 | 182.6 | 22.2 | 96.48 |
| **III** | **(8)** | 39.17 | 172.36 | 29.49 | 174.7 | 7.88 | 130.59 |
| **IV** | **(9)** | 46.22 | 166.74 | 32.28 | 176.1 | 1.44 | - |
| **V** | **(6)** | 33.82 | 191.13 | 5.56 | 185.4 | 35.04 | 98.11 |
| **VI** | **(7)** | 8.88 | 170.59 | 26.9 | 178.8 | 28.39 | 60.51 |
| **VII** | **(2)** | 21.05 | 177.13 | 37.1 | 181.96 | 34.88 | 71.82 |

**Entries I-IV:** Equimolar blends with PLA of opposite chirality to the functionalized copolymer; **Entries V-VI:** Equimolar blends with identical thiol-functionalized copolymers of opposite lactide chirality; **Entry VII**: Equimolar blend with **Polymer (6)**; DSC analysis conditions: 5 mg samples, N_2_ atmosphere, 10 °C min^-1^ heating and cooling rates; ^a^*T*_g_ at the 2^nd^ heating scan; ^b^Melting temperature measured by DSC analysis in the first heating run; ^c^Samples obtained by solution crystallization; ^d^Melting temperature measured by DSC analysis in the second heating run; ^e^Results obtained from samples in the melt.

# References

1. Coulembier, O.; Sanders, D. P.; Nelson, A.; Hollenbeck, A. N.; Horn, H. W.; Rice, J. E.; Fujiwara, M.; Dubois, P.; Hedrick, J. L., Hydrogen-Bonding Catalysts Based on Fluorinated Alcohol Derivatives for Living Polymerization. *Angew. Chem. Int. Ed.* **2009,** *48* (28), 5170-5173.

2. Lohmeijer, B. G. G.; Pratt, R. C.; Leibfarth, F.; Logan, J. W.; Long, D. A.; Dove, A. P.; Nederberg, F.; Choi, J.; Wade, C.; Waymouth, R. M.; Hedrick, J. L., Guanidine and amidine organocatalysts for ring-opening polymerization of cyclic esters. *Macromolecules* **2006,** *39* (25), 8574-8583.

3. Venkataraman, S.; Ng, V. W. L.; Coady, D. J.; Horn, H. W.; Jones, G. O.; Fung, T. S.; Sardon, H.; Waymouth, R. M.; Hedrick, J. L.; Yang, Y. Y., A Simple and Facile Approach to Aliphatic N-Substituted Functional Eight-Membered Cyclic Carbonates and Their Organocatalytic Polymerization. *J. Am. Chem. Soc.* **2015,** *137* (43), 13851-13860.

4. Bexis, P.; De Winter, J.; Arno, M. C.; Coulembier, O.; Dove, A. P., Organocatalytic Synthesis of Alkyne-Functional Aliphatic Polycarbonates via Ring-Opening Polymerization of an Eight-Membered-N-Cyclic Carbonate. *Macromol Rapid Commun* **2021,** *42* (3), e2000378.

5. Makiguchi, K.; Kikuchi, S.; Yanai, K.; Ogasawara, Y.; Sato, S.-i.; Satoh, T.; Kakuchi, T., Diphenyl phosphate/4-dimethylaminopyridine as an efficient binary organocatalyst system for controlled/living ring-opening polymerization of L-lactide leading to diblock and end-functionalized poly(L-lactide)s. *J. Polym. Sci. Part A: Polym. Chem.* **2014,** *52* (7), 1047-1054.

6. Nederberg, F.; Connor, E. F.; Möller, M.; Glauser, T.; Hedrick, J. L., New Paradigms for Organic Catalysts: The First Organocatalytic Living Polymerization. *Angew. Chem. Int. Ed.* **2001,** *40* (14), 2712-2715.

7. Tüdos, F.; Kelen, T.; Földes-berezsnich, T.; Turcsányi, B., Analysis of Linear Methods for Determining Copolymerization Reactivity Ratios. III. Linear Graphic Method for Evaluating Data Obtained at High Conversion Levels. *J. Macrom. Sci. A: Chem.* **2006,** *10* (8), 1513-1540.

8. Fineman, M.; Ross, S. D., Linear method for determining monomer reactivity ratios in copolymerization. *J. Polym. Sci.* **1950,** *5* (2), 259-262.

9. Campos, J. M.; Ribeiro, M. R.; Ribeiro, M. F.; Deffieux, A.; Peruch, F., Copolymerisation of ε-caprolactone and trimethylene carbonate catalysed by methanesulfonic acid. *Eur. Polym. J.* **2013,** *49* (12), 4025-4034.

10. Gnanou, Y.; Fontaine, L., *Organic and Physical Chemistry of Polymers*. John Wiley & Sons, Inc.: 2008.

11. Tsuji, H., Poly(lactide) Stereocomplexes: Formation, Structure, Properties, Degradation, and Applications. *Macromol. Biosci.* **2005,** *5* (7), 569-597.

12. Xie, Z.; Hu, X.; Chen, X.; Sun, J.; Shi, Q.; Jing, X., Synthesis and Characterization of Novel Biodegradable Poly(carbonate ester)s with Photolabile Protecting Groups. *Biomacromolecules* **2008,** *9* (1), 376-380.

13. Liu, X.; Hua, X.; Cui, D., Copolymerization of Lactide and Cyclic Carbonate via Highly Stereoselective Catalysts To Modulate Copolymer Sequences. *Macromolecules* **2018,** *51* (3), 930-937.

14. Dobrzynski, P.; Kasperczyk, J., Synthesis of biodegradable copolymers with low‐toxicity zirconium compounds. V. Multiblock and random copolymers of L‐lactide with trimethylene carbonate obtained in copolymerizations initiated with zirconium(IV) acetylacetonate. *J. Polym. Sci. Part A: Polym. Chem.* **2006,** *44* (10), 3184-3201.

15. Fox, T. G.; Flory, P. J., The glass temperature and related properties of polystyrene. Influence of molecular weight. *J. Polym. Sci.* **1954,** *14* (75), 315-319.

16. Fox, T. G.; Flory, P. J., Second‐Order Transition Temperatures and Related Properties of Polystyrene. I. Influence of Molecular Weight. *J. Appl. Phys.* **1950,** *21* (6), 581-591.

17. Gordon, J. M.; Rouse, G. B.; Gibbs, J. H.; Risen, W. M., The composition dependence of glass transition properties. *J. Chem. Phys.* **1977,** *66* (11), 4971-4976.

18. Yuen, A. Y.; Bossion, A.; Veloso, A.; Mecerreyes, D.; Hedrick, J. L.; Dove, A. P.; Sardon, H., Efficient polymerization and post-modification of N-substituted eight-membered cyclic carbonates containing allyl groups. *Polym. Chem.* **2018,** *9* (18), 2458-2467.

19. Socka, M.; Duda, A.; Adamus, A.; Wach, R. A.; Ulanski, P., Lactide/trimethylene carbonate triblock copolymers: Controlled sequential polymerization and properties. *Polymer* **2016,** *87*, 50-63.

20. Thomas, A. W.; Kuroishi, P. K.; Pérez-Madrigal, M. M.; Whittaker, A. K.; Dove, A. P., Synthesis of aliphatic polycarbonates with a tuneable thermal response. *Polym. Chem.* **2017,** *8* (34), 5082-5090.
